# *CaHsfA1d* Improves Plant Thermotolerance via Regulating the Expression of Stress- and Antioxidant-Related Genes

**DOI:** 10.3390/ijms21218374

**Published:** 2020-11-08

**Authors:** Wen-Xian Gai, Xiao Ma, Yang Li, Jing-Jing Xiao, Abid Khan, Quan-Hui Li, Zhen-Hui Gong

**Affiliations:** 1 College of Horticulture, Northwest A&F University, Yangling 712100, China; gaiwenxian@nwafu.edu.cn (W.-X.G.); mxiao26@163.com (X.M.); 18002429961@163.com (Y.L.); jingjingxiao136@163.com (J.-J.X.); 2 Department of Horticulture, The University of Haripur, Haripur 22620, Pakistan; abidagriculturist@gmail.com; 3 Academy of Agricultural and Forestry Sciences, Qinghai University, Xining 810016, China

**Keywords:** Hsf, heat stress, H_2_O_2_, virus-induced gene silencing (VIGS), overexpression, seed germination

## Abstract

Heat shock transcription factor (Hsf) plays an important role in regulating plant thermotolerance. The function and regulatory mechanism of *CaHsfA1d* in heat stress tolerance of pepper have not been reported yet. In this study, phylogenetic tree and sequence analyses confirmed that *CaHsfA1d* is a class A Hsf. *CaHsfA1d* harbored transcriptional function and predicted the aromatic, hydrophobic, and acidic (AHA) motif mediated function of *CaHsfA1d* as a transcription activator. Subcellular localization assay showed that *CaHsfA1d* protein is localized in the nucleus. The *CaHsfA1d* was transcriptionally up-regulated at high temperatures and its expression in the thermotolerant pepper line R9 was more sensitive than that in thermosensitive pepper line B6. The function of *CaHsfA1d* under heat stress was characterized in *CaHsfA1d*-silenced pepper plants and *CaHsfA1d*-overexpression *Arabidopsis* plants. Silencing of the *CaHsfA1d* reduced the thermotolerance of the pepper, while *CaHsfA1d*-overexpression *Arabidopsis* plants exhibited an increased insensitivity to high temperatures. Moreover, the *CaHsfA1d* maintained the H_2_O_2_ dynamic balance under heat stress and increased the expression of *Hsfs*, *Hsps* (heat shock protein), and antioxidant gene *AtGSTU5* (glutathione S-transferase class tau 5) in transgenic lines. Our findings clearly indicate that *CaHsfA1d* improved the plant thermotolerance via regulating the expression of stress- and antioxidant-related genes.

## 1. Introduction

Environmental factors, especially heat stress, can significantly threaten crop productivity and grain quality worldwide. In many farming regions of the world, yield of major crops are likely to be seriously affected as global warming worsens [1]. Continuous high temperatures can cause drought which may damage the water balance of the plants, leading to wilting and desiccation. In addition, the heat stimulus has significant adverse effects on plant growth due to peroxidation of the membrane system [2]. Being a sessile organism, plants are constantly exposed to environmental stresses and unable to escape from high environmental temperature. In order to survive, plants have formed a series of adaptive physiological mechanisms and molecular regulatory networks to respond to these rising temperatures and improve their own resistance in the long evolutionary process. It has become clear that early perception and regulatory networks of heat shock signal in plants are mainly mediated by transcriptional factors [3]. To date, many important genes modulating thermotolerance have been recognized in plants. Heat shock transcription factors (Hsfs) act as main regulatory components and key terminal factors of the heat shock response during the signal transduction pathway of heat stress, and can regulate the expression of downstream target genes related to defense against environmental stresses [4,5,6,7].

Hsfs are found in many eukaryotes. Only one single Hsf member is identified in *Drosophila melanogaster*, *Caenorhabditis elegans*, and yeast, while four Hsf genes are found in the vertebrate genome [8,9,10,11,12]. In contrast, the Hsf family usually contains dozens of members among the examined plants so far. The number of Hsfs in plants is more than that in other organisms. HSF genes found in *Arabidopsis* (*Arabidopsis thaliana*) are 21 [13], 25 in rice (*Oryza sativa*) [13], 24 in tomato (*Solanum lycopersicum*) [14], 56 in wheat (*Triticum aestivum*) [15], and 30 in maize (*Zea mays* L.) [6]. Numerous Hsfs in plants may form a more complex regulatory network to improve the tolerance to heat stress. The structure of Hsf protein is conservatively modular in the plant. Plant Hsfs contain a DNA binding domain (DBD), specifically recognizing and binding to the heat stress elements (HSEs) in the promoter regions of the downstream genes, the oligomerization (HR-A/B) harboring the heptad hydrophobic repeats, and the intracellular nuclear localization signal (NLS) [4,6,14,16]. Some Hsfs also contain nuclear export signals (NES) [14,17]. Based on the number of amino acids inserted between the HR-A/B domains, plant Hsfs can be divided into three subfamilies (HsfA, B, and C) [17]. Class HsfAs also have the aromatic, hydrophobic, and acidic (AHA) domain at the C-terminus, but class HsfBs and HsfCs do not contain these domains. Thus, HsfAs become a transcriptional activator serving to regulate the heat stress response in plants due to the presence of the AHA domain [8,15]. Unlike HsfAs, HsfBs does not contain AHA domains, resulting in their lack of transcriptional activity. A repressor domain (RD) is located at the C-terminus of the HsfB protein, and it is speculated that it functions as a repressor motif, so that HsfB members can act as repressors [18]. HsfC may play an active role in regulating plant heat tolerance, and its positive effect may be related to the induction or up-regulation of heat-resistant genes [19]. In Addition, the Hsf members in vertebrates can also act as developmental regulators and are important for lifespan-enhancing pathways [12].

Many studies divulged that HsfA1s play important roles in regulating plant thermotolerance (Table 1). Four HsfA1s, i.e., HsfA1a, HsfA1b, HsfA1c, and HsfA1e, exist in *Arabidopsis*. The thermotolerance of *HsfA1a* and *HsfA1b* double-knockout mutant are significantly impaired in *Arabidopsis* [20], whereas overexpression of *HsfA1a* or *HsfA1b* enhance the expression levels of heat shock protein (Hsp) genes under normal conditions and the basal thermotolerance of *Arabidopsis* plants [21,22]. The *Arabidopsis* Hsp90 can interact with HsfA1 to inhibit the accumulation of HsfA1 protein in the nucleus [23]. In addition, the *HsfA1a*/*HsfA1b*/*HsfA1d*/*HsfA1e* quadruple-knockout *Arabidopsis* mutant display extremely weakened basal thermotolerance (BT) and acquired thermotolerance (AT) [24], and it is also proposed that the thermotolerance enhanced by exogenous melatonin is largely alleviated in this mutant and HsfA1s may be involved in melatonin-mediated thermotolerance in *Arabidopsis* [25]. Meanwhile, the *HsfA1a*/*HsfA1b*/*HsfA1d* are also involved in thermotolerance to mild heat stress [26]. Thus, these results suggest that *HsfA1s* may play key functions as regulators of heat stress response in *Arabidopsis*. Tomato also has four *HsfA1* genes, namely *HsfA1a*, *HsfA1b*, *HsfA1d*, and *HsfA1e*, in which *HsfA1* was reported to be a master regulator of heat stress response [27]. Meanwhile, the expression of *HsfA1a* is constitutive under control and stress conditions, while the other members are induced in specific tissues and stages of heat stress response [28]. *HsfA1s* are early heat stress response genes activating the expression of the late response *Hsf* genes such as *HsfA2* in *Arabidopsis* as well as in tomato [24,29]. The physical interaction between HsfA1 and heat stress-inducible HsfA2 can form activator heterodimers, resulting in the transactivation activity of target heat stress genes expression [30]. Most studies of *HsfA1s* are restricted to model plants, but there are also some studies of *HsfA1s* in other species [1,8,31,32]. Overexpression of the Lily (*Lilium longiflorum*) *LiHsfA1* gene improves the thermotolerance of transgenic *Arabidopsis* and up-regulates the expression of putative stress-response genes [31]. *GmHsfA1*, encoding a novel and functional Hsf, has been characterized from soybeans (*Glycine max*), and transgenic soybeans with its overexpression showed obviously enhance thermotolerance under heat stress [1]. In maize (*Zea mays*), two HsfA1 members (ZmHsf06 and ZmHsf12) are identified [32] and heat tolerance of the *Arabidopsis* seedlings overexpressed with *ZmHsf06* or *ZmHsf12* was increased [8,33].

Except for HsfA1s, other members of subfamily HsfA are also found to positively regulate the induction of thermotolerance in various plants. A heat-inducible transcription factor, HsfA2, is required for extending the duration of AT in *Arabidopsis* [29]. *HsfA3*, which is under the control of DREB2A (dehydration-responsive element binding protein), is shown to play an important role in thermotolerance of *Arabidopsis* and maize [34,35,36]. HsfA4s are potent activators of heat stress gene expression and HsfA5 may represent a specific repressor to regulate the function of HsfA4s in plant [37]. The transcriptional activity of the *HsfA6b* promoter can be activated by the AREB1 (ABA-responsive element-binding protein 1), then HsfA6b and AREB1 activate DREB2A expression in concert to mediate a complex regulatory network of the heat stress response [38].

Pepper (*Capsicum annuum* L.) is an economically important vegetable crop and sensitive to heat stress [18,39]. Hsfs acts as an important regularly factor in response to heat stress, but its function is poorly understood in pepper. Although we have identified 25 pepper *Hsf* (*CaHsf*) genes in previous work and analyzed the expression of *CaHsfA2* after heat stress treatment [18,39], more work is still needed to be performed in order to further explore the functions of *Hsfs* and its molecular mechanism in pepper thermotolerance. The 17 *CaHsfA* genes are found in these pepper *CaHsfs* and *CaHsfA1d*, *CaHsfA2*, and *CaHsfA3* are the main *CaHsfA* members with significantly higher expression levels under heat stress, suggesting that these *CaHsfAs* may be the key transcription factors in response to thermal stress [18]. In the current study, we have quantitatively analyzed the expression of the *CaHsfA1d* gene in different tissues of pepper as well as under heat stress. The subcellular localization and transactivation activity of CaHsfA1d protein were also determined. The *CaHsfA1d*-silenced pepper plants and *CaHsfA1d*-overexpression *Arabidopsis* plants were generated to analyze the function and regulatory mechanism of *CaHsfA1d* in response to heat stress. It was concluded that *CaHsfA1d* could improve the plant thermotolerance via maintaining H_2_O_2_ homeostasis.

## 2. Results

### 2.1. Isolation and Characterization Analyses of CaHsfA1d

Recently, Hsfs have been found to be noteworthy regulators in modifiable plant thermotolerance. To predict and analyze the function of pepper *CaHsfA1d*, we cloned the *CaHsfA1d* gene from two pepper lines, the thermotolerant pepper line R9 (introduced from the World-Asia Vegetable Research and Development Center, PP0042-51) and the thermosensitive pepper line B6 (selected by the pepper research group, College of Horticulture, Northwest A&F University, Yangling, China) [40]. Through cDNA sequence comparison of the *CaHsfA1d* in the R9 and B6 pepper line, it was concluded that the nucleotide sequence of 1509 bp CDS was completely the same in the two varieties. The *CaHsfA1d* was predicted to encode 502 amino acid residues of 55.62 kDa with an isoelectric point of 4.65.

**Figure 1:**
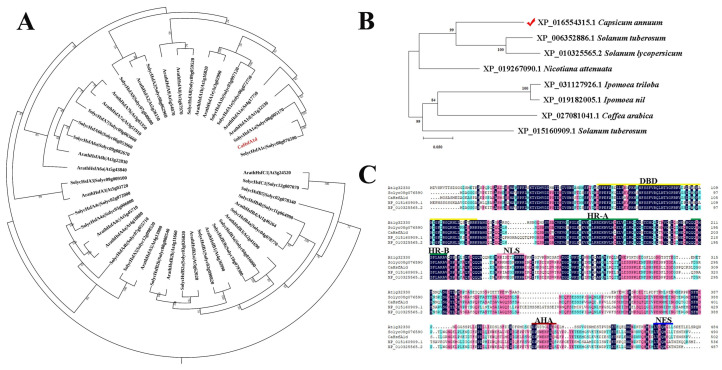
Sequence analysis of the *CaHsfA1d* protein. (**A**) Phylogenetic relationship of the *CaHsfA1d* with *Arabidopsis* (ArathHsfs) and tomato (SolycHsfs) Hsf proteins. The Hsf proteins of the *Arabidopsis* and tomato were downloaded from the Heatster (http://www.cibiv.at/services/hsf/). The neighbor-joining (NJ) phylogenetic tree was constructed using MEGA-X (http://www.megasoftware.net/) with the following parameters: Poisson model, bootstrap (1000 replicates), and pairwise deletion gaps. (**B**) Phylogenetic relationship of *CaHsfA1d* (XP_016554315.1) and Hsfs from other plant species. The protein sequence of *CaHsfA1d* was used to perform the BLASTP search in the database NCBI Protein Reference Sequences (https://blast.ncbi.nlm.nih.gov/). Eight HSF proteins were selected to construct the NJ phylogenetic tree by MEGA-X using the parameters in (**A**). (**C**) Multiple sequence alignment of Hsf proteins. Clustal Omega software (http://www.clustal.org/) was used to align the Hsf sequences using default parameters and the results were minor repaired by DNAMAN Version 9.0 (www.lynnon.com) software. The domains have been indicated. At1g32330 (*Arabidopsis thaliana*, ArathHsfA1d), Solyc08g076590 (*Solanum lycopersicum*, SolycHsfA1c), XP_015160909.1 (*Solanum tuberosum*), XP_010325565.2 (*Solanum lycopersicum*).

Phylogenetic relationship analysis of CaHsfA1d with 21 *Arabidopsis* Hsfs and 24 tomato Hsfs showed that CaHsfA1d belongs to the subfamily of HsfA1 (Figure 1A). CaHsfA1d was also clustered into a group together with ArathHsfA1a, ArathHsfA1d, SolycHsfA1a, and SolycHsfA1c (Figure 1A). Additionally, the protein sequence of *CaHsfA1d* was used to perform the BLASTP search in database of NCBI Protein Reference Sequences. Eight Hsfs closely related to the *CaHsfA1d* protein were downloaded and one of the obtained proteins was *CaHsfA1d* protein numbered by XP_016554315.1 in NCBI (Figure 1B). An additional phylogenetic tree was subsequently constructed to analyze the evolutional relationship of all acquired Hsfs from different plant species, including *Capsicum annuum*, *Nicotiana attenuate*, *Solanum tuberosum*, *Solanum lycopersicum*, *Ipomoea triloba*, *Ipomoea nil*, and *Coffea arabica* (Figure 1B). *CaHsfA1d* exhibited a close relationship to two putative proteins, i.e., XP_006352886.1 in *Solanum tuberosum* and XP_010325565.2 in *Solanum lycopersicum.* Multiple sequence alignment indicated that the *CaHsfA1d* protein shared highest similarity with Hsfs from *Arabidopsis*, *Solanum tuberosum*, and *Solanum lycopersicum*. The alignment confirmed that the *CaHsfA1d* contained the typical domains of HsfA1, including a conserved DBD, a HR-A/B domain inserted by 21 amino acids, a NLS, a NES, and an AHA motif (Figure 1C). These data suggested that *CaHsfA1d* was a member of HsfA1 in pepper.

### 2.2. Transactivation Activity Analysis of the CaHsfA1d Protein

**Figure 2:**
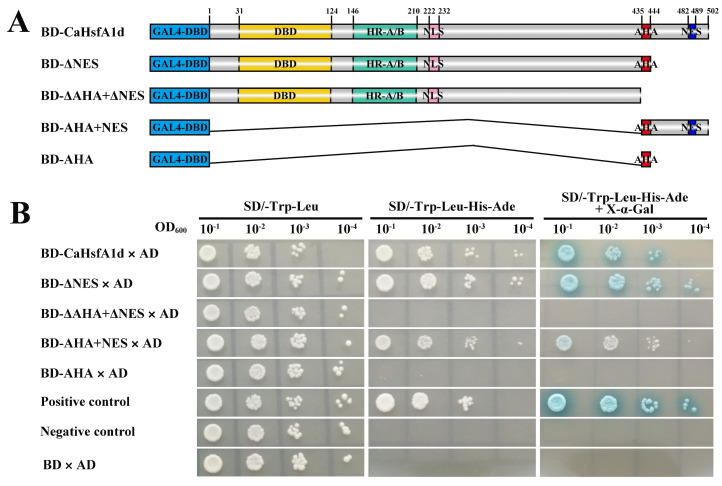
Transactivation assay of the *CaHsfA1d* protein in yeast cells. (**A**) Schematic representation of the full-length *CaHsfA1d* and deletion protein constructs in the pGBKT7 vector. (**B**) Growth of transformed yeast cells 3 days after spotting on selective mediums. Positive control, yeast cells transformed with pGBKT7-53 and pGADT7-T; Negative control, yeast cells transformed with pGBKT7-Lam and pGADT7-T. Experiments were performed three times with similar results.

To detect the transcriptional function of the *CaHsfA1d* as a transcription factor in pepper, we performed the yeast two-hybrid procedure to evaluate its transactivation activity. One complete and four deletion sequences of the *CaHsfA1d* protein were constructed in frame with the GAL4-DBD to obtain translational fusion with GAL4-DBD (BD-*CaHsfA1d*, BD-ΔNES, BD-ΔAHA+ΔNES, BD-AHA+NES, and BD-AHA) (Figure 2A). As shown in Figure 2B, all transgenic yeasts grew well on SD/-Trp-Leu medium. The yeast cells harboring the BD-*CaHsfA1d* plasmid grew as well as the positive control on the SD/-Trp-Leu-His-Ade medium and exhibited positive for α-Gal activity on SD/-Trp-Leu-His-Ade+X-α-Gal medium, suggesting that the *CaHsfA1d* protein had transactivation activity to act as a transcriptional activator. Compared with the full *CaHsfA1d* protein, the deletion mutant BD-ΔNES of *CaHsfA1d* showed equal transactivation activity and the BD-ΔAHA+ΔNES mutant exhibited no activity in yeast. This result indicated that the AHA motif could play an important role in activating function. The fragment BD-AHA+NES still indicated weak activity, while the fragment BD-AHA showed no activation in yeast. Moreover, the BD-ΔAHA+ΔNES mutant growing on the selective medium for 5 days (Figure S1) displayed stronger transactivation activity than that growing for 3 days (Figure 2B). The fragment BD-AHA actually exhibited a weak transactivation activity in nutrition deficiency screening and α-Gal staining assays (Figure S1). It was evident that *CaHsfA1d* harbored transcriptional function and the predicted AHA motif was important for its transactivation activity.

### 2.3. Subcellular Location of the CaHsfA1d Protein in Tobacco

**Figure 3:**
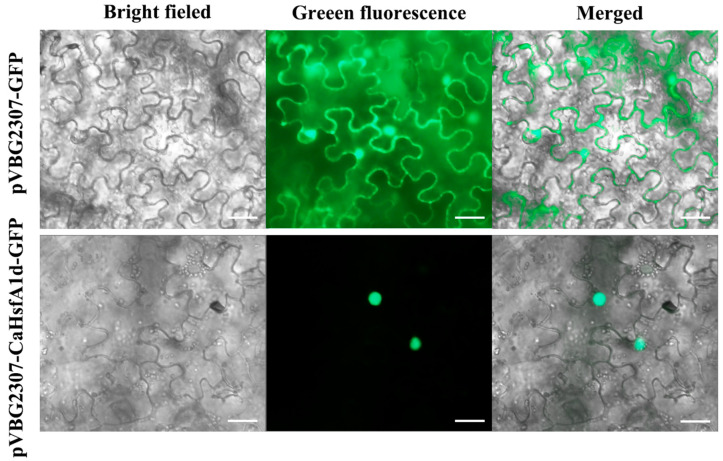
Subcellular localization of the CaHsfA1d protein in *Nicotiana benthamiana*. The scale bars represent 25 µm.

To examine the subcellular localization, the *CaHsfA1d* protein fused with GFP at the C-terminal side was driven by a CaMV 35S promoter in pVBG2307-GFP vector. A plasmid containing GFP alone was used as a control. After transient expression with *Agrobacterium*-mediated transformation in tobacco, green fluorescence of the *CaHsfA1d*-GFP protein could only be detected in the nucleus, while the control GFP signals were uniformly distributed throughout the whole cell (Figure 3). Thus, it indicated that *CaHsfA1d* protein was only located in the nucleus.

### 2.4. Expression Analyses of CaHsfA1d in Different Tissues and Response to Heat Stress

**Figure 4:**
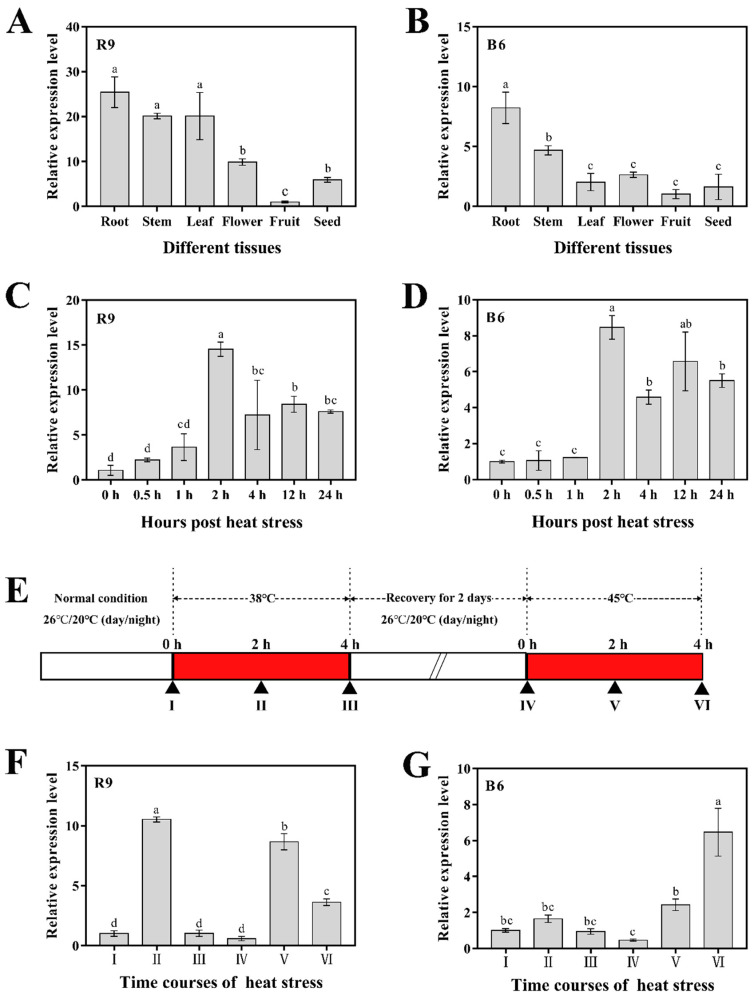
The *CaHsfA1d* expression in different tissues and response to heat treatment. (**A**,**B**) Tissue-specific expression levels of *CaHsfA1d* in pepper line R9 or B6. The expression level in fruit was set as 1. (**C**,**D**) The *CaHsfA1d* expression analysis in pepper BT in the R9 or B6 leaves. (**E**) Schematic representation of the heat stress regimes of Figure 4F,G. (**F**,**G**) The *CaHsfA1d* expression analysis in pepper AT in the R9 or B6 leaves. Pepper *CaUBI3* (ubiquitin-conjugating protein-coding gene) is used as a reference gene to normalize the transcript levels of *CaHsfA1d* upon different samples. The expression at 0 h is set as 1. Data are means ±SD of three biological replicates. Different lowercases letters denote statistically significant differences from the control treatment at *p* ≤ 0.05 by *t*-test.

To investigate the expression pattern of the *CaHsfA1d* gene, we analyzed the expression levels of six pepper tissues under normal growth conditions and response to its BT and AT treatments in the R9 or B6 leaves (Figure 4). Tissue-specific expression showed that *CaHsfA1d* was variably expressed in all tissues, including root, stem, leaf, flower, fruit, and seed (Figure 4A,B). The highest expression level was detected in the roots of both R9 and B6 seedlings, while the lowest was found in fruits. Compared with the expression in various tissues of thermosensitive line B6 (Figure 4B), *CaHsfA1d* was expressed at relatively higher levels in thermotolerance line R9 (Figure 4A). In particular, the *CaHsfA1d* transcripts were abundant in the leaves of R9 and relatively poor in the same tissue of B6. For the BT analysis, *CaHsfA1d* was strongly up-regulated in the leaves of both R9 and B6 under the 45 °C temperature (Figure 4C,D). The transcription level in R9 (Figure 4C) was shown to be more rapidly and powerfully expressed in response to temperature compared with that in B6 (Figure 4D). Compared with the control, the *CaHsfA1d* expression was induced to the highest level (14.5 and 8.45-fold increase in R9 and B6, respectively) in the both materials at 2 h post heat stress and then kept at a high expression level even at 24 h post heat stress. All R9 samples exhibited higher expression levels than those in B6. Likewise, for the AT analysis (Figure 4F,G), the transcriptional expression of *CaHsfA1d* displayed a significantly different pattern between R9 and B6. The *CaHsfA1d* expression in R9 was strongly increased at the 38 °C treatment stage, decreased at the 22 °C recovery stage, and highly increased at 45 °C. However, *CaHsfA1d* could not be induced in thermosensitive seedlings B6 under the temperature 38 °C, while its expression was only induced under the 45 °C treatment.

The results indicated that the expression level of *CaHsfA1d* varied in different tissues and response to heat treatments between the thermotolerance pepper R9 and thermosensitive pepper B6, implying that the transcriptional regulation of the *CaHsfA1d* gene played a potential role in the induction of thermotolerance.

### 2.5. Performance of the CaHsfA1d-Silenced Pepper under Heat Stress

The induction of *CaHsfA1d* expression by heat stress pointed to its involvement in high temperature responsiveness. To test the possible role of *CaHsfA1d* in pepper thermotolerance, *CaHsfA1d* was silenced in the R9 pepper line to examine its effect in response to heat stress. The fragment of *CaHsfA1d* was inserted into the TRV2 vector in order to silence the *CaHsfA1d* in pepper plants through virus-induced gene silencing (VIGS) [41,42]. The SGN VIGS tool was used to avoid off-target silencing potential. The newly grown leaves of plants transformed with *TRV2:CaPDS* showed the photo-bleaching phenotype (Figure S2A), while no visible phenotypic differences were observed between *TRV2:CaHsfA1d* and *TRV2:00* pepper plants under normal conditions. Subsequently, the silencing efficiency of *TRV2:00* and *TRV2:CaHsfA1d* was assessed using the specific qRT-PCR primers (Figure S2B). Compared with the control (*TRV2:00*), *CaHsfA1d* was expressed at a level approximately 4.05-folds lower in *CaHsfA1d*-silenced plants, demonstrating that *CaHsfA1d* had been successfully silenced via VIGS and could be used for further studies.

**Figure 5:**
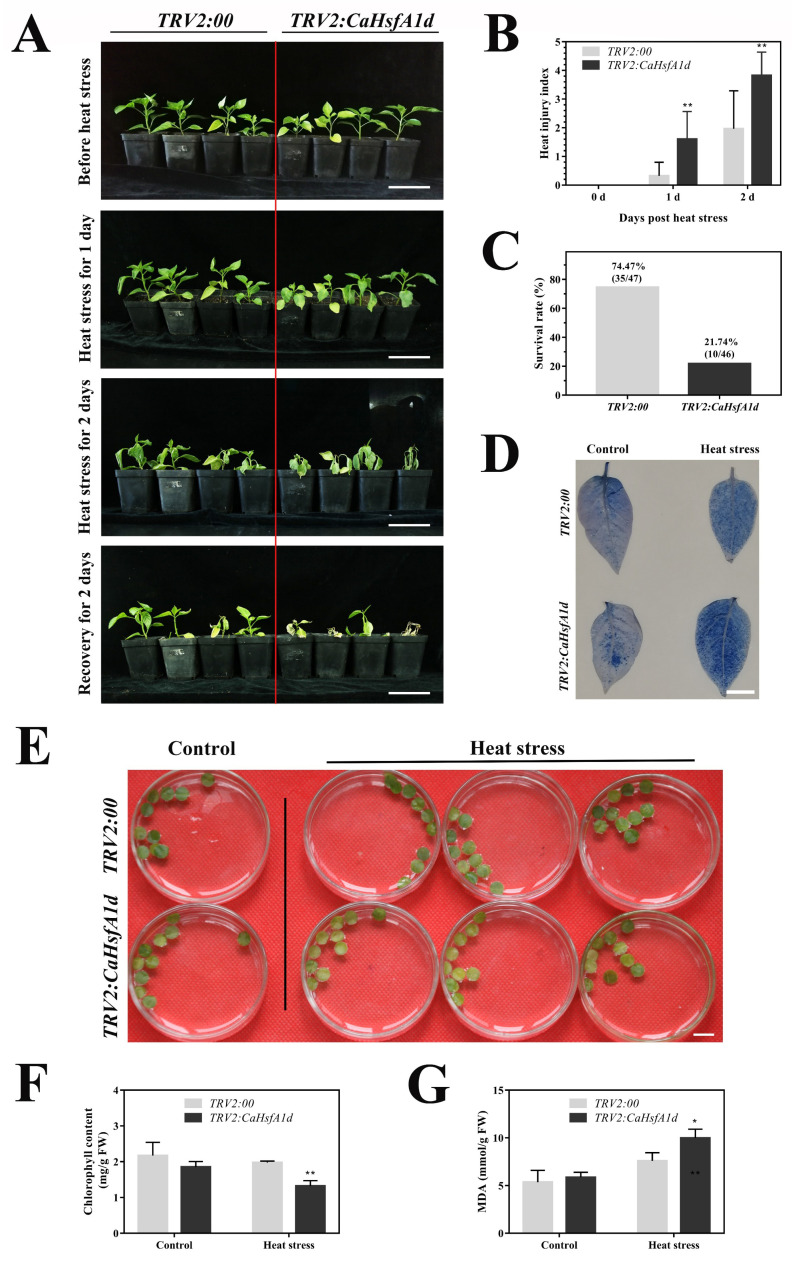
Thermotolerance analyses of *CaHsfA1d*-silenced pepper seedlings. (**A**) Appearance of *TRV2:00* and *TRV2:CaHsfA1d* pepper plants before heat treatment, after heat treatment, and after recovery. Scale bar = 10 cm. (**B**) Heat injury index scored daily for *TRV2:00* and *TRV2:CaHsfA1d* pepper plants after heat treatment in Figure 5A. (**C**) Survival rate of *TRV2:00* and *TRV2:CaHsfA1d* seedlings after recovery in Figure 5A. (**D**) Trypan blue coloration in the leaves of *TRV2:CaHsfA1d* pepper plants compared with *TRV2:00* after 45 °C treatment for 2 h. Scale bar = 1 cm. (**E**) The phenotype of leaf discs isolated from *TRV2:00* or *TRV2:CaHsfA1d* plants at 12 h post 37 °C heat stress. Scale bar = 1 cm. (**F**) Chlorophyll and (**G**) MDA content of the leaf discs (Figure 5E) exposed to heat stress. Data are means ± SD of three biological replicates. The asterisks on the top of bars indicate significant differences between the *TRV2:00* and *TRV2:CaHsfA1d* plants. * *p* < 0.05, ** *p* < 0.01 by *t*-test.

The heat tolerance of the control and the *CaHsfA1d*-silenced seedlings were assessed upon heat treatment (Figure 5). After heat treatment under 42/38 °C day/night, more serious symptoms of leaves wilting were observed in the *CaHsfA1d*-silenced plants as compared to the *TRV2:00* plants at 1- or 2-day high temperature condition, while no obvious differences appeared before treatment (Figure 5A). *CaHsfA1d*-silenced plants had a higher heat injury index compared with *TRV2:00* plants (Figure 5B). During a 2-day-recovery period of 22/18 °C day/night following heat stress treatment, most of wilted leaves in the *CaHsfA1d*-silenced pepper plants became yellow and numerous plants died because of high temperature. The survival rate of *CaHsfA1d*-silenced plants was only 21.74% (10/46 live/dead), while that of *TRV2:00* plants was 74.47% (35/47 live/dead) (Figure 5C). In addition, the trypan blue stain assay was carried out to examine the cell death in leaves grown under optimal temperature or heat stress at 45 °C for 2 h (Figure 5D). The results demonstrated that more intense trypan blue coloration was observed in the *TRV2:CaHsfA1d* leaves than that of *TRV2:00*. Moreover, the excised leaf discs isolated from the *TRV2:00* and *TRV2:CaHsfA1d* plants were exposed to 42 °C for 12 h (Figure 5E). Subsequently, the chlorophyll (Figure 5F) and MDA (Figure 5G) contents of the leaf discs were measured. The *CaHsfA1d*-silenced plants exhibited weaker thermotolerance, and their chlorophyll and MDA contents were lower as compared to the *TRV2:00* plants. These results indicated that silencing of the *CaHsfA1d* reduced the thermotolerance of the pepper.

### 2.6. Thermotolerance Analyses of the CaHsfA1d-Overexpression Arabidopsis

**Figure 6:**
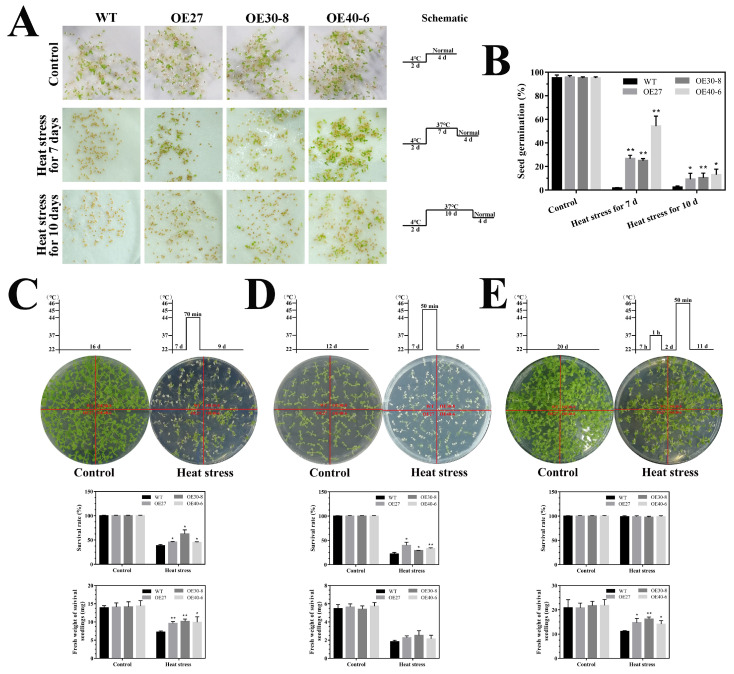
Thermotolerance analyses of *CaHsfA1d*-overexpression *Arabidopsis* seeds and seedlings. (**A**) The germination phenotypes and (**B**) germination rates of transgenic and WT seeds grown at 22 °C for 4 days after preheating at 37 °C for 7 or 10 days. Schematics at the right of Figure 6A show the heat stress regimes of *Arabidopsis* seeds. (**C**–**E**) Performance of the *CaHsfA1d*-overexpression and WT seedlings under heat stress. Schematics at the top of Figure 6C–E show the heat stress regimes of *Arabidopsis* seedlings. Data are means ±SD of three biological replicates. The asterisks on the top of bars indicate significant differences between *CaHsfA1d* transgenic and WT plants. * *p* < 0.05, ** *p* < 0.01 by *t*-test. Scale bar = 1 cm.

To confirm and further investigate the results of the *CaHsfA1d*-silencing experiments, we successfully generated the *CaHsfA1d*-overexpression transgenic *Arabidopsis* and confirmed the transcript levels of the *CaHsfA1d* by semi-quantitative RT-PCR and qRT-PCR (Figure S2C,D). Three independent *CaHsfA1d*-overexpression lines (OE27, OE30-8, and OE40-6), which displayed differential expressions (Figure S2C,D), were selected to perform the basal and acquired thermotolerance assays. No significant differences in the seed germination, seedling growth, and development of the transgenic *Arabidopsis* and wild type (WT) plants were observed under normal growth conditions (Figure 6). After *Arabidopsis* seeds were exposed to 37 °C for 7 or 10 days, the WT seeds hardly germinated with optimal growth conditions for 4 days (Figure 6A,B). In contrast, the *CaHsfA1d* transgenic seeds have higher germination rates in BT and AT heat processing modes during a recovery period of 4 days (Figure 6A,B). The result suggested that overexpressing the *CaHsfA1d* gene could markedly improve the thermotolerance of *Arabidopsis* seeds. Basal-thermotolerance of *CaHsfA1d*-overexpressing seedlings were evaluated upon 44 or 45 °C treatment for 70 or 50 min, respectively (Figure 6C,D). Upon two heat stress treatment regimens, the transgenic *Arabidopsis* lines exhibited stronger basal thermotolerance as compared to the WT. The fresh weight of survived seedlings and the survival rates after heat stress were evidently higher in *CaHsfA1d*-overexpression lines compared to WT plants. Next, we compared the growth status of overexpression and WT seedlings when exposed to 46 °C after preheating at 37 °C (Figure 6E). No matter under normal growth conditions or heat treatment, there were no obvious differences in the survival rates between overexpression lines and WT; however, under heat stress, the *CaHsfA1d*-overexpressed plants showed higher fresh weight than WT. Preheating of the seedlings significantly increased the survival rates of both overexpression and WT plants, but extreme temperature still prejudiced the growth situations of *Arabidopsis*. Therefore, these results confirmed that overexpression of the *CaHsfA1d* increased the resistant of seeds and plants to heat stress.

### 2.7. Association between CaHsfA1d and H_2_O_2_ Accumulation in Heat Stress

The accumulation and degradation of H_2_O_2_ in plants are balanced in the natural growth environment. However, various environmental stresses frequently result in a burst of H_2_O_2_, ensuing in damage to the plant cell [43,44]. The cell death in pepper leaves was detected at high temperature and a much higher level of cell death was observed in *CaHsfA1d-silenced* leaves as compared to the control (Figure 5D). We supposed that the cell death might be due to the imbalance of H_2_O_2_ in pepper leaves and the silencing of the *CaHsfA1d* gene increased H_2_O_2_ accumulation triggering more severe cell death (as revealed by trypan blue staining).

**Figure 7:**
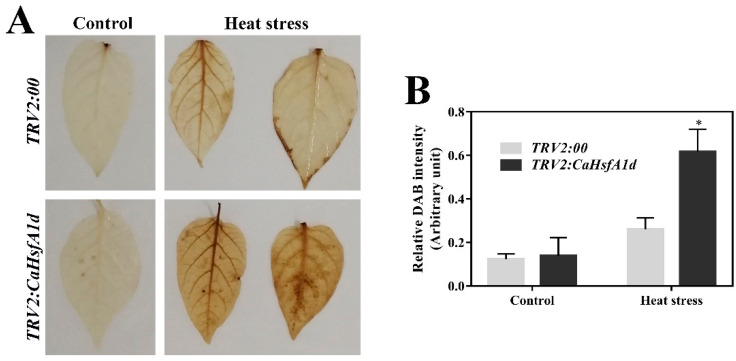
Detection of H_2_O_2_ in the control and silenced pepper leaves. (**A**) H_2_O_2_ staining from leaves of *TRV2:00* and *TRV2:CaHsfA1d* plants post 45 °C heat treatment for 2 h. (**B**) Quantification of relative DAB staining of the control and silenced pepper. Data are means ± SD of three biological replicates. The asterisks on the top of bars indicate significant differences between the *TRV2:00* and *TRV2:CaHsfA1d* plants. * *p* < 0.05 by *t*-test. Scale bar = 1 cm.

**Figure 8:**
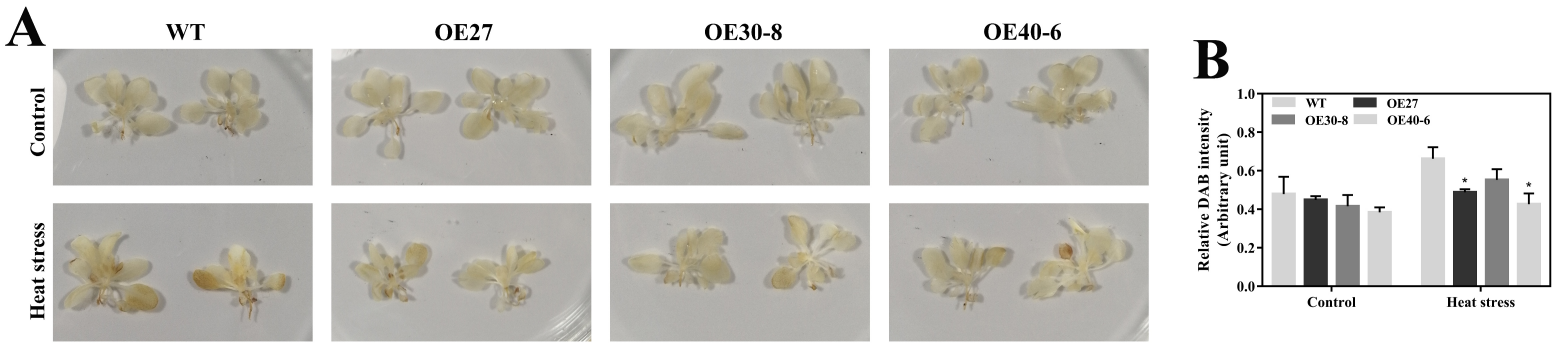
Detection of H_2_O_2_ in the leaves of WT and overexpression *Arabidopsis* lines. (**A**) H_2_O_2_ staining from the leaves of WT and transgenic plants post 37 °C heat treatment for 2 h. (**B**) Quantification of relative DAB staining of the WT and *CaHsfA1d*-overexpression *Arabidopsis*. Data are means ± SD of three biological replicates. The asterisks on the top of bars indicate significant differences between WT and overexpressing lines. * *p* < 0.05 by *t*-test. Scale bar = 1 cm.

To test this possibility, H_2_O_2_ accumulation in response to heat stress were compared between the control and *CaHsfA1d*-silenced leaves with the histochemical staining of 3, 3′-diaminobenzidine (DAB) (Figure 7A,B). The relative accumulation of H_2_O_2_ in control seedlings upon heat stress was 2.12 times higher than that without heat treatment. After heat stress, strong H_2_O_2_ accumulation was noted in the absence of *CaHsfA1d* and much higher production of H_2_O_2_ was triggered in the *CaHsfA1d*-silenced leaves as compared to the control plants. To further explore these results, the accumulation of H_2_O_2_ in the WT and *CaHsfA1d-*overexpression lines were distinguished at 2 h heat stress (Figure 8A,B). Consistently, leaves of overexpressing *Arabidopsis* lines showed a darker yellow color (Figure 8A) and had a higher H_2_O_2_ content as compared to the WT leaves (Figure 8B). These results indicated that exposure to heat stress triggered the H_2_O_2_ accumulation and *CaHsfA1d* played a significant role in maintaining the balance of the H_2_O_2_ production and decomposition under heat stress.

### 2.8. Expression Analyses of the Stress-Related Genes in Transgenic Arabidopsis

**Figure 9:**
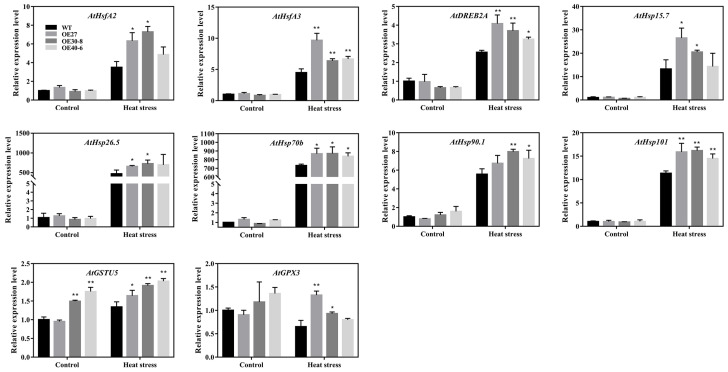
Expression of stress-related genes in *Arabidopsis*. The gene expression levels of 12-day-old *Arabidopsis* seedlings treated under 37 °C for 0 or 2 h were detected by qRT-PCR. All primers are shown in Table S1. Data are means ±SD of three biological replicates. The asterisks on the top of bars indicate significant differences between *CaHsfA1d* transgenic and WT plants. * *p* < 0.05, ** *p* < 0.01 by *t*-test. *AtHsfA2* (At2G26150), *AtHsfA3* (At5G03720), *AtDREB2A* (At5g05410), *AtHsp15.7* (At5G37670), *AtHsp26.5* (At1G52560), *AtHsp70b* (At1G16030), *AtHsp90.1* (At5G52640), *AtHsp101* (At1G74310), *AtGSTU5* (At2G29450), *AtGPX3* (At2G43350).

Our data showed that overexpression of *CaHsfA1d* decreased sensitivity to heat stress in *Arabidopsis*, and that silencing of the *CaHsfA1d* enhanced susceptibility to heat stress in pepper (Figure 5 and Figure 6). Since we have evidenced the transcriptional function of *CaHsfA1d* protein (Figure 2), the downstream genes conferring heat sensitivity were detected by qRT-PCR in transgenic and WT *Arabidopsis* (Figure 9). After heat stress treatment, the mRNA levels of two transcriptional factor genes (*AtHsfA2* and *AtDREB2A*) displayed stronger induction in overexpressing plants than in WT, whereas the expression levels of the *AtHsp70b*, *AtHsp90.1*, and *AtHsp101* were also more induced in the transgenic plants than in the WT. Additionally, no significant difference in the expression of the autochthonous *AtHsfA1d* gene was detected in all *Arabidopsis* lines (Figure S3). Specifically, we found that the role of *CaHsfA1d* as a positive regulator of plant thermotolerance was also associated with H_2_O_2_ accumulation (Figure 7 and Figure 8). We reasoned that *CaHsfA1d* could participate in the progress of scavenging ROS and CaHsfA1d might act as a crucial transcriptional factor to regulate the expression of antioxidant genes under heat stress. To test this hypothesis, the transgenic and WT *Arabidopsis* were used to measure the expression of antioxidant enzyme-related genes including superoxide dismutase (*AtSOD*), catalase (*AtCAT*), ascorbic acid peroxidases (*AtAPX1* and *AtAPX2*), as well as glutathione antioxidant-related genes (*AtGSTU5* and *AtGPX3*) (Figure S3 and Figure 9). Only *AtGSTU5* and *AtGPX3* were up-regulated in *CaHsfA1d*-overexpression lines as compared with WT (Figure 9); while the expression of the antioxidant enzyme genes (*AtSOD*, *AtCAT*, *AtAPX1*, and *AtAPX2*) exhibited no significant difference in the transgenic and WT *Arabidopsis* upon exposure to excess heat stress (Figure S3). These results suggested that overexpression of the *CaHsfA1d* gene enhanced the expression of *AtHsfA2*/*3*, *AtDREB2A*, and *AtHsps* genes as well as glutathione synthesis related genes (*AtGSTU5* and *AtGPX3*) in transgenic plants.

## 3. Discussion

Although plant heat tolerance has been intensively studied in recent decades and Hsf proteins have been implicated in response to this process [9,25,30,33,37,45,46], however, the function and regulatory mechanism of CaHsfA1d was less characterized in pepper. In current study, the function of a heat shock transcription factor protein, pepper CaHsfA1d, was analyzed and characterized in pepper.

Phylogenetic tree and sequence analyses confirmed that the CaHsfA1d was a class A Hsf. The evolution tree from different plant species exhibited that CaHsfA1d had a closest relationship with *solanaceous* plants (Figure 1B). Multiple sequence alignment indicated that the CaHsfA1d protein included completely conserved motifs with high similarity to those in *Arabidopsis* and *Solanum* plants (Figure 1C). In these identified conserved motifs, a short AHA domain located at C-terminus and mediated the function of HsfAs as transcription activator [6]. Previous study has shown that Trp and Leu located in AHA motif might play serious character on the transactivation activity of AtHsfA [47]. Moreover, *Lilium longiflorum* LlHsfA2b lacked transcriptional function due to the absence of Trp and Leu in its separate AHA motif [48]. The AHA of CaHsfA1d protein evidently possessed the feature by multiple sequence alignment (Figure 1C). Thus, the CaHsfA1d activation mediated by just one AHA was analyzed in yeast activation assay (Figure 2). The results showed that the AHA motif was essential for CaHsfA1d transcriptional activity. Interestingly, the AHA of *Arabidopsis* HsfA1d (At1g32330), being the homology protein of CaHsfA1d, has no a complete and typical AHA. This might suggest that pepper *CaHsfA1d* harbored more specific function as compared to *Arabidopsis* HsfA1d. *CaHsfA1d* was also nucleus protein (Figure 3), which is in agreement with the result of LlHSFA1 [31]. The putative NLS motif in *CaHsfA1d* also indicated that *CaHsfA1d* would probably be recognized by the NLS receptor for its nuclear localization [31,49]. For heat shock transcriptional factor protein, the transcriptional activation via AHA and the nuclear localization via NLS are necessary to regulate the expression of their downstream genes [50].

The expression patterns of the thermotolerant pepper line R9 and the thermosensitive pepper line B6 were compared (Figure 4). The results showed that the transcripts levels of the *CaHsfA1d* was detected in various pepper tissues, and it was highly expressed in R9 than in B6 (Figure 4A,B). In particular, the expression level of the *CahsfA1d* in R9 leaves was at a level approximately 10-fold higher than that in B6, implying that a large number of differential expressions might affect the agronomic characteristics of pepper. The heat-induced expression pattern is a feature of known HsfA1 members [9,23,24,25,31,51]. As expected, the *CaHsfA1d* expression was induced under BT and AT heat treatments in leaves and displayed stronger expression level in R9 (Figure 4C–G). It was interesting to find that there seemed to be a close relationship between the thermal resistance of pepper and the expression levels of *CaHsfA1d* gene. Taken together, *CaHsfA1d* might be involved in response to heat stress in pepper and its abundant transcript levels might play potential role in the induction of thermotolerance.

In pepper, the *CaHsfA1d* was induced by high temperature, signifying its role in response to excess heat exposure. To clarify this role of the *CaHsfA1d*, the *CaHsfA1d*-silenced pepper plants and *CaHsfA1d*-overexpression *Arabidopsis* lines were generated. After heat stress treatment, it was noticed that the chlorophyll and MDA contents of the silenced pepper plant leaf discs were substantially lower than the control plant leaf discs (Figure 5E–G), and the lower survival rate and higher heat-injury index were presented in *CaHsfA1d*-silenced plants (Figure 5A–C). In addition, *CaHsfA1d*-silenced leaves exhibited more sensitivity to heat as revealed by darker trypan blue staining compared with control plants when challenged with extreme heat (Figure 5D). Moreover, *CaHsfA1d*-overexpression *Arabidopsis* plants exhibited an increased insensitivity to high temperature (Figure 6), having higher survival rates and fresh weight than WT after heat treatment. Together, our findings showed that *CaHsfA1d* was a positive regulator of heat tolerance in pepper. Many studies presented in the introduction of this article supported our findings [3,4,5,6,7,46]. It has been reported that overexpression of *GmHsfA1* in soybean showed an obvious enhancement of thermotolerance compared with non-transgenic plants [51].

Although the effects of *Hsf* in response to heat stress have been previously reported, still little is known about the regulatory mechanism of heat stress responses in pepper. When pepper plants were exposed to heat, the *CaHsfA1d* was highly expressed in the initial stressful period. It could be postulated that *CaHsfA1d* might play a leading role at the front end of the signal path in the transference of the heat signaling. Many studies have given an explicit support for that conclusion. For example, several Hsfs (HsfA1, HsfA7, and HsfBs etc.) were located downstream of HsfA1 in the thermal signal network and HsfA1 activated and regulated the expression of *Hsfs* [9,23,30,31]. Furthermore, HsfA1a/b/d/e in response to environmental stress regulated the expression levels of heat stress related genes including the dominant factor *HsfA2*, *HsfA7a/b* as well as *DREB2A* and its downstream gene *HsfA3* [38]. Similarly, under heat stress, *Arabidopsis* HsfA1d/HsfA1e have not only activated *HsfA2* transcription through the HSE elements but also induced other *Hsf* genes (*HsfA7a*, *HsfA7b*, *HsfB1*, and *HsfB2a*) expression [9,35]. At the same time, the transcript levels of *AtHsfA2* and *AtHsfA3* in the *CaHsfA1d*-overexpression *Arabidopsis* were higher than those in WT with or without heat stress (Figure 9), while compared with the *TRV2:00* plants, the transcript levels of the pepper *CaHsfA2* and *CaHsfA3* genes showed weaker induction in the *CaHsfA1d*-silenced leaves under heat stress (Figure S4). These evidences might propose that overexpression of the *CaHsfA1d* increased the expression levels of *AtHsfA2* and *AtHsfA3* in transgenic *Arabidopsis* and silencing of the *CaHsfA1d* decreased the expression levels of *CaHsfA2* and *CaHsfA3* in pepper. Moreover, overexpression of *HsfA2* or *HsfA3* conferred increased thermotolerance in transgenic *Arabidopsis* [52,53,54]. Then we could speculate that *CaHsfA1d* improved the plant thermotolerance by up-regulating the expressions of *AtHsfA2* and *AtHsfA3* genes.

To our knowledge, *Hsps* are known target genes of Hsfs, and Hsf are able to activate its expression by binding to the HSEs in their promoter regions [4,6,24]. The *Hsps* are present in all plant species and act vital roles in enhancing plant thermotolerance and alleviating heat stress damage to plants [44,55,56,57,58,59]. In pepper, two pepper members of cytosolic Hsp70 functioned as molecular chaperone proteins and played an important role in response to plant stress [60,61]. *CaHsp70-1* was involved in heat stress defense via Ca^2+^ signal transduction pathway [61], while *CaHsp70-2* conferred improved thermotolerance in *Arabidopsis* by regulating expression of stress-related genes [60]. In addition, the overexpression of pepper *CaHsp16.4* or *CaHsp25.9* improved the plant thermotolerance under heat stress [44,56]. Interestingly, our results just exhibited that the expression of the two pepper genes were significantly decreased in the *CaHsfA1d*-silenced pepper under heat stress, which caused loss of the pepper thermotolerance (Figure S4). In *Arabidopsis*, *AtHsp101* was induced by heat stress and involved in the acquired thermo-tolerance [44,62]. As expected, in our study, we noticed that the expression levels of *AtHsps* were significantly up-regulated in the *CaHsfA1d*-overexpression *Arabidopsis* compared to those of WT seedlings after heat stress treatment (Figure 9). This result proved that CaHsfA1d-activated transcripts of *Hsps* contributed to the plant thermotolerance.

Reactive oxygen species (ROS) including H_2_O_2_ is a hampering factor for plant growth and development caused by environment stresses. The results of the current study indicated that exposure to heat stress triggered the H_2_O_2_ accumulation in plants (Figure 7 and Figure 8). Under heat stress, the enhanced H_2_O_2_ accumulation might be due to the absence of *CaHsfA1d* in *CaHsfA1d*-silenced peppers, while overexpressing the *CaHsfA1d* in *Arabidopsis* has significantly decreased the H_2_O_2_ accumulation as compared to WT. To defend plant cells from oxidative injury, plants have developed various detoxification systems including ascorbic acid, water-soluble reductants, glutathione, as well as antioxidant enzymes involving ROS scavengers, such as POD, CAT, and APX [63,64]. Previous studies have strongly demonstrated the connection between antioxidant enzymes and Hsfs in scavenging ROS [63,65,66]. For instance, *Arabidopsis* Hsf3 was involved in modulating the expression of *APX2* to scavenge H_2_O_2_ [67]. The similar pattern between ROS and Hsps has also been recognized to protect plants from oxidative stress [44,56]. However, the expression levels of these antioxidant genes including *AtSOD1*, *AtCAT1*, *AtAPX1*, and *AtAPX2* exhibited no significant difference in all lines detected (Figure S3). Interestingly the *AtGSTU5* and *AtGPX3* genes were strongly up-regulated in *CaHsfA1d*-overexpression lines compared with WT (Figure 9). The enzymes glutathione-S-transferase (GST) and glutathione peroxidase (GPX) use glutathione as a substrate to scavenge H_2_O_2_ [68] and *AtGSTU5* and *AtGPX3* are involved in the glutathione antioxidant system [56,64,69]. *AtGSTU5* accumulated exposed to various environmental stresses in plants [70] and participated in the scavenging of H_2_O_2_ [64]. *PuHSFA4a* increased GST activity for reducing ROS acumination by directly binding to the promoter of *PuGSTU17* in *Populus ussuriensis* [68]. Additionally, the mutant of *Arabidopsis AtGPX3* exhibited higher sensitivity to H_2_O_2_ treatment and *ATGPX3* might function as a general scavenger in H_2_O_2_ homeostasis [69]. All evidences have emerged to suggest that *CaHsfA1d* activated the expression of ROS-scavenging genes *AtGSTU5* and *AtGPX3* to improve plant thermotolerance.

**Figure 10:**
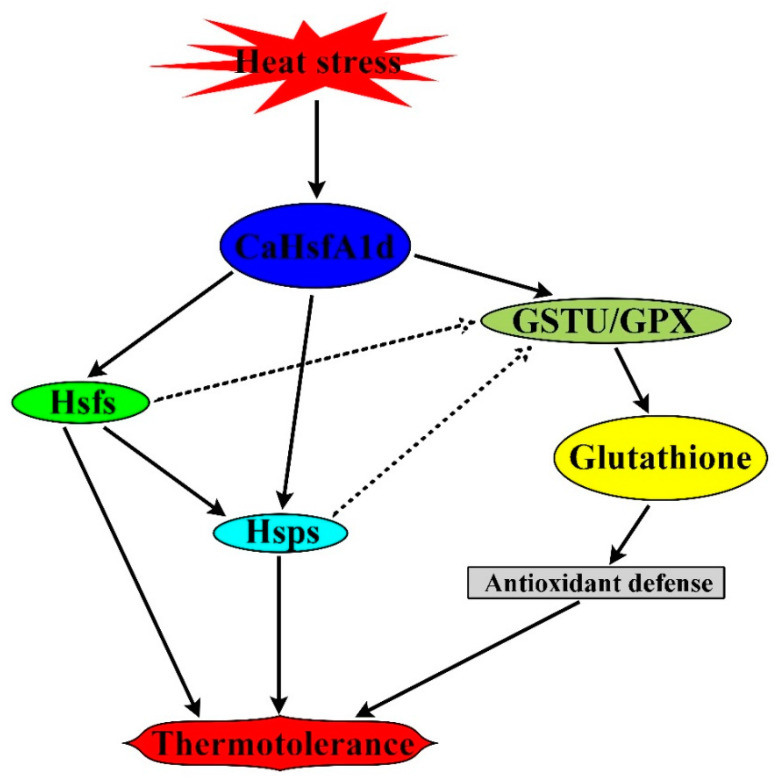
Working model proposed for CaHsfA1d-mediated thermotolerance in plant.

Based on these findings, we propose a possible working model depicting the mechanism of *CaHsfA1d*-mediated thermotolerance in plant (Figure 10). When heat stress was applied, the *CaHsfA1d* transcript was highly and quickly induced. Then, CaHsfA1d protein activated the expression of heat-responsive genes, including not only *Hsfs* and *Hsps* with enhancing the plant thermotolerance, but also *GSTU* and *GPX* participating in the detoxification of H_2_O_2_ through using glutathione as a substrate to eliminate ROS. Furthermore, we speculate that Hsfs and Hsps may also be partially involved in the regulation of *GSTU* and *GPX*. The results in this study contribute to point the important role of pepper *CaHsfA1d* gene in improving the plant thermotolerance.

## 4. Materials and Methods

### 4.1. Plant Materials and Growth Conditions

Two pepper lines R9 and B6 were used to analyze the expression patterns and the line R9 was also used for VIGS in this study. Additionally, we used the WT *A. thaliana* (ecotype Columbia) to construct the transgenic plants. All seedlings were grown in environment-controlled growth chambers with 60 to 70% relative humidity under a 16 h day/8 h night regime. The temperature varied depending on different experiments. The pepper plants were grown at 25/18 °C day/night to analyze the gene expression patterns. The R9 pepper line was grown at 22/18 °C day/night for VIGS experiment. The temperature was also set to 22/20 °C day/night for *Arabidopsis* seedlings.

### 4.2. Cloning and Expression Pattern of the CaHsfA1d Gene

The full-length sequence of *CaHsfA1d* was amplified from R9 and B6 pepper leaves cDNA using the CaHsfA1d-clone primers (Table S1). The amplified fragments were cloned in pMD19-T (TaKaRa, Dalian, China) and sequenced.

To analyze the expression profiles of *CaHsfA1d* in pepper, we performed the tissue-specific expression analysis and the analyses in response to heat stress. For tissue-specific expression analysis, the R9 and B6 pepper seedlings were grown in a natural glasshouse with the temperature 25 to 29 °C/16 to 20 °C (day/night). Samples from roots, stems, leaves, flower buds, fruits, and seeds were collected to analyze the expression levels of *CaHsfA1d.* For expression analysis of the *CaHsfA1d* in pepper R9 and B6 lines under heat stress, the plants at 6–8 true leaves stage were exposed to 45 °C temperature to analyze the role in BT, and then the third leaves of the seedlings were harvested at different hours post heat treatment. We also analyzed the expression of *CaHsfA1d* in response to AT in peppers at 6–8 true leaves. The third leaves at different treatment stages were collected and stored, respectively. The experiment was carried out in three biological replicates, each containing six seedlings. All collected samples were immediately frozen in liquid nitrogen and stored at −80 °C fridge until RNA extraction.

### 4.3. Transactivation Activity Assay in Yeast

The transcriptional activity of the CaHsfA1d protein was analyzed with the yeast two-hybrid assay according to the manufacturer’s introduction (Clontech, CA, USA). The full CDS of CaHsfA1d and the derived deletion fragments were amplified from pepper line R9 using the corresponding primers as listed in Table S1. Subsequently, all PCR products were inserted between the BamH I and Pst I sites of the GAL4-DBD vector pGBKT7. The fusion-construction plasmids were then transformed into the yeast strain Y2HGold together with the pGADT7 empty vector, respectively. The positive transgenic strains were picked into liquid SD/-Trp-Leu medium and the cultures were shaken at 30 °C until the OD600 was 0.8. The yeast cells were collected by centrifugation and diluted to OD600 of 10^−1^, 10^−2^, 10^−3^, and 10^−4^ with 0.9% NaCl. The resulting yeasts dropped onto selective mediums (SD/-Trp-Leu, SD/-Trp-Leu-His-Ade, and SD/-Trp-Leu-His-Ade+X-α-Gal) at 30 °C for 3–5 days.

### 4.4. Subcellular Localization in Tobacco

To observe the subcellular localization of CaHsfA1d protein, we generated a pVBG2307-CaHsfA1d-GFP vector with GFP protein fused at the C-terminal of the CaHsfA1d protein. The open reading frame (ORF) of CaHsfA1d without a termination codon was amplified using the pepper line R9 cDNA with the pVBG2307-CaHsfA1d-GFP primers (Table S1). The obtained vector pVBG2307-CaHsfA1d-GFP and the control empty vector pVBG2307-GFP were separately transformed into *Agrobacterium tumefaciens* strain GV3101. Subsequently, the *Agrobacterium*-mediated transient gene expression assay was performed to identify the subcellular localization of CaHsfA1d protein with the previous method [71]. The fluorescence signal in tobacco epidermis was detected using a fluorescence microscope BX63 (Olympus Corporation, Tokyo, Japan).

### 4.5. The Thermotolerance Analyses in the CaHsfA1d-Silenced Pepper

To assess the role of *CaHsfA1d* in pepper thermotolerance, the tobacco rattle virus-based VIGS was used to silence the *CaHsfA1d* gene in pepper. A specific 365 bp fragment of *CaHsfA1d* was obtained from the pepper line R9 using TRV2-CaHsfA1d primers designed by the SGN VIGS Tool (https://vigs.solgenomics.net/) (Table S1). The PCR product was inserted into the TRV2 vector to generate the *TRV2:CaHsfA1d*-silencing vector. The blank vector *TRV2:00* was taken as the control, while the *TRV2:CaPDS* (Phytoene desaturase gene) was taken as a phenotype for successful gene silencing assay. Briefly, the *CaHsfA1d*-silenced pepper plants (R9 line) were generated following the method as described in our previous study [42]. The *CaHsfA1d*-silenced pepper plants were used to distinguish the thermotolerance.

The pepper plants containing *TRV2:00* and *TRV2:CaHsfA1d* were incubated at the regime 42/38 °C (day/night) for 2 days and kept the soil moist at all times, then allowed to recover for 2 days under normal conditions (22/18 °C day/night). The heat injury index was scored on daily basis, as follows: 0 (no wilting), 1 (less wilting), 2 (partial wilting), 3 (wilting), and 4 (severe wilting or dead). The survival rates of *TRV2:00* and *TRV2:CaHsfA1d* seedlings were also measured after two-day recovery, respectively. To compare H_2_O_2_ accumulation and cell death of pepper leaves under high temperature, both *TRV2:00* and *TRV2:CaHsfA1d* seedlings were treated under 45 °C for 2 h, and the control plants were mock-treated with normal temperature. The leaves from both treated and control plants were collected to perform the DAB and trypan blue staining according to the previously described method [72,73]. The quantification of DAB was obtained following the method by Chakraborty et al. [72]. The excised leaf discs (0.5 cm in diameter) from the *TRV2:00* and *TRV2:CaHsfA1d* plants were also treated at 42 °C in pure water for 12 h. The MDA and chlorophyll contents of each treatment were determined with the previously described methods [74,75].

### 4.6. The Thermotolerance Analyses in the CaHsfA1d-Overexpression Arabidopsis

pVBG2307-*CaHsfA1d* vector with the CaMV35S promoter was combined with the *CaHsfA1d* fragment amplified from R9 cDNA by pVBG2307-CaHsfA1d-GFP primers (Table S1). The *CaHsfA1d*-overexpression vector was transformed into the *Agrobacterium tumefaciens* GV3101 strain using the freeze–thaw transformation method. Subsequently, the transformation of *Arabidopsis* was performed by the floral dip method [76]. All harvested *Arabidopsis* seeds were screened on 1/2 MS medium complemented with 50 mg/L kanamycin and then seven stable lines of transgenic *Arabidopsis* were obtained after several generations of continuous screening. The *CaHsfA1d* transcript of seven positive transgenic lines were further confirmed by semi-quantitative RT–PCR and qRT-PCR using specific primers (Figure S2C,D, Table S1). The T4 seeds of transgenic line OE27, OE30-8, and OE40-6 were used for experimental analysis. Additionally, all the overexpression and WT *Arabidopsis* seeds were harvested at the same time.

To detect the effect of overexpressing *CaHsfA1d* in the *Arabidopsis* seeds thermotolerance, three transgenic lines and WT were placed in airtight petri dishes lined with wet filter paper, respectively. All seeds were treated by exposure to dark conditions at 4 °C for 2 days and then maintained in an environment-controlled chamber at 37 °C in darkness for 7 or 10 days for heat stress. After heat treatment, all petri dishes having *Arabidopsis* seeds were transferred into a growth chamber with a normal growing condition (22/18 °C day/night). The unheated-stress transgenic or WT *Arabidopsis* seeds grown under the normal growth conditions were used as positive control. The germination rates of all treatments were measured after grown 4 days at normal environment.

For heat stress treatments on *CaHsfA1d-*overexpression *Arabidopsis*, we performed three independent experiments with the WT and transgenic *Arabidopsis*. Two assays were implemented to analyze the effect on basal thermotolerance of overexpressing *CaHsfA1d Arabidopsis*. Seven-day-old *Arabidopsis* seedlings were exposed to 44 °C or 45 °C for 70 or 50 min, respectively and then correspondingly recovered for 9 days or 5 days under normal growth conditions. Assay of acquired thermotolerance were also carried out with the seven-day-old *Arabidopsis* based on the previously described method [16]. The *Arabidopsis* seedlings were preheated at 37 °C for 60 min, recovered under normal growth conditions for 2 days before treated for 60 min at 46 °C temperature, and then recovered for another 11 days. The survival rates and the fresh weight of survival seedlings were measured and representative photographs were taken to record viability. The experiments were performed with three biological replicates. To analyze the H_2_O_2_ accumulation of *Arabidopsis* under heat stress, WT and *CaHsfA1d*-overexpression plants were exposed to 37 °C for 2 h, and the control seedlings were treated under normal conditions. The DAB staining and quantification of DAB was followed as previously described methods [72,73].

### 4.7. RNA Isolation and qRT-PCR Analyses

RNA extraction was performed with all collected samples using the RNA extraction kit (Tiangen, Shanghai, China). The attained RNA samples were then reverse-transcribed to first-strand cDNA by the PrimeScript^TM^ RT reagent Kit with the gDNA Eraser (TaKaRa, Beijing, China) according to the manufacturer’s protocol. qRT-PCR was completed using SYBR^®^ Premix Ex Taq™ II (TaKaRa, Beijing, China). *Arabidopsis ATACT2* and pepper *CaUBI3* (ubiquitin-conjugating protein-coding gene) genes were used for normalization of *Arabidopsis* and pepper, respectively. All primers used in this study are listed in Supplementary Table S1.

## Figures and Tables

**Table 1:** Summary of plant *HsfA1* involved in thermotolerance.

Gene	Plant	Function	Reference
*HsfA1a*/*HsfA1b*	*Arabidopsis*	The thermotolerance of *HsfA1a* and *HsfA1b* double-knockout mutant are significantly impaired. Overexpression of *HsfA1a* or *HsfA1b* enhanced the thermotolerance.	[20,21,22]
*HsfA1*	*Arabidopsis*	*Arabidopsis* Hsp90 can interact with HsfA1 to inhibit the accumulation of HsfA1 protein in the nucleus.	[23]
*HsfA1a*/*HsfA1b*/*HsfA1d*/*HsfA1e*	*Arabidopsis*	The quadruple-knockout *Arabidopsis* mutant display extremely weakened BT and AT. *HsfA1s* may be involved in melatonin-mediated heat tolerance.	[24,25]
*HsfA1a*/*HsfA1b*/*HsfA1d*	*Arabidopsis*	The *HsfA1a*/*HsfA1b*/*HsfA1d* are also involved in thermotolerance to mild heat stress.	[26]
*HsfA1a*	*Solanum lycopersicum*	The expression of *HsfA1a* is constitutive under control and stress conditions.	[28]
*HsfA1*	*Solanum lycopersicum*	The physical interaction between HsfA1 and heat stress-inducible HsfA2 can form activator heterodimers, resulting in the transactivation activity of target heat stress genes expression.	[30]
*LiHsfA1*	*Lilium longiflorum*	Overexpression of *LiHsfA1* gene improves the thermotolerance of transgenic. *Arabidopsis* and up-regulated the expression of putative stress-response genes.	[31]
*GmHsfA1*	*Glycine max*	The transgenic soybeans with its overexpression showed obviously enhance thermotolerance under heat stress.	[1]
*ZmHsf06*/*ZmHsf12*	*Zea mays*	The heat tolerance of the *Arabidopsis* seedlings overexpressed with *ZmHsf06* or *ZmHsf12* was increased.	[8,33]

## Supplementary Materials

Supplementary Materials can be found at https://www.mdpi.com/1422-0067/21/21/8374/s1.
