# Supplementary material for: CaHsfA1d Improves Plant Thermotolerance via Regulating the Expression of Stress- and Antioxidant-Related Genes
Source: Int J Mol Sci. 2020 Nov 8;21(21):8374. doi: 10.3390/ijms21218374 (PMC7672572; doi:10.3390/ijms21218374)
Supplement: Supplementary file 1 [file ijms-21-08374-s001.zip › sup/Supplementary Material.docx]

Supplementary Material


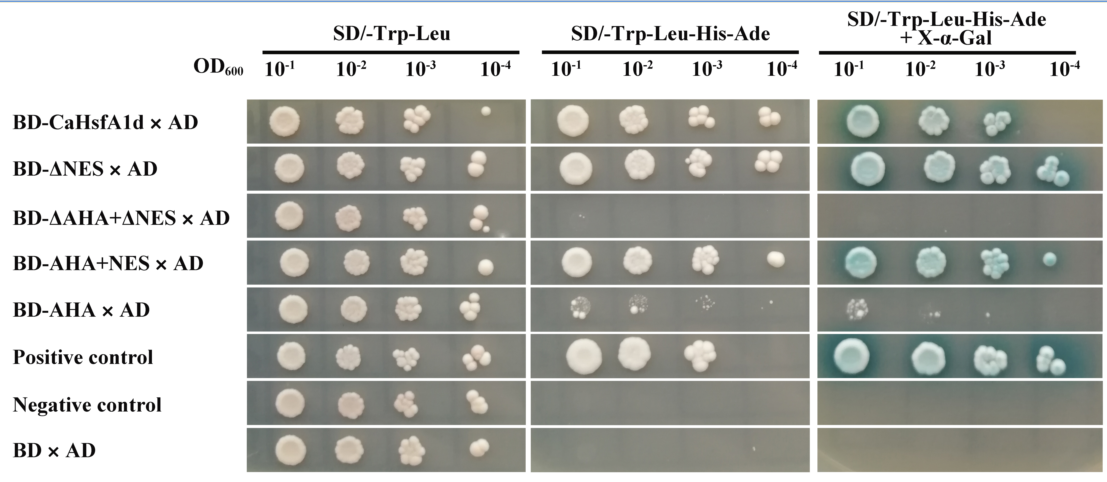


**Figure S1.** Growth of transformed yeast cells 5 days after spotting on selective mediums. Positive control, yeast cells transformed with pGBKT7-53 and pGADT7-T; Negative control, yeast cells transformed with pGBKT7-Lam and pGADT7-T. Experiments were performed three times with similar results.


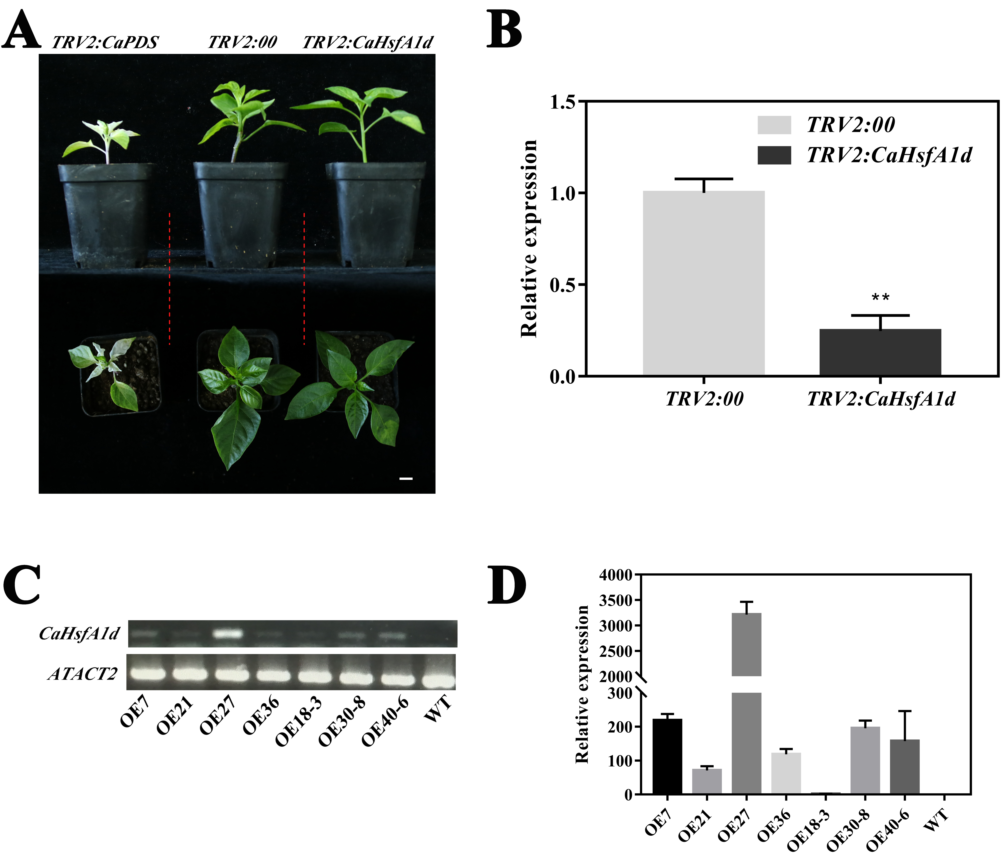


**Figure S2.** Confirmation of the *CaHsfA1d*-silenced pepper and *CaHsfA1d*-overexpressing *Arabidopsis*. (A) Phenotypes of the pepper plants 21 d after inoculation. *TRV2:PDS*, seedlings with the *TRV2:PDS* vector (PDS, phytoene desaturase gene for chlorophyll synthesis); *TRV2:00*, control plants with the black TRV2 vector; *TRV2:CaHsfA1d*, *CaHsfA1d*-silenced seedlings. Scale represents 1 cm. (B) The efficiency of gene expression silencing in *CaHsfA1d*-silenced peppers. qRT-PCR was performed to analyze the expression with the specific qRT-PCR primer (Table S1). The *CaUBI3* gene was used as a reference. ∗∗ indicates significant difference at the 0.01 levels by *t*-test. (C) Semi-quantitative RT–PCR analysis of transcript levels of *CaHsfA1d* in the wild-type and overexpression *Arabidopsis*. The primers sqRT-PCR-CaHsfA1d were used for amplification and *ATACT2* gene was used as the internal control. (D) Determination of the *CaHsfA1d* expression level in *CaHsfA1d*-overexpressing *Arabidopsis* lines by qRT-PCR. Specific primers qRT-PCR-CaHsfA1d were used for qRT-PCR. Relative transcript levels were normalized to *ATACT2* mRNA. No value of the *CaHsfA1d* expression was obtained in WT and the value of the OE18-3 plants was set equal to 1. WT, wide type *Arabidopsis*; OE, Arabidopsis transgenic lines with *CaHsfA1d* gene. All primers are listed in Table S1.


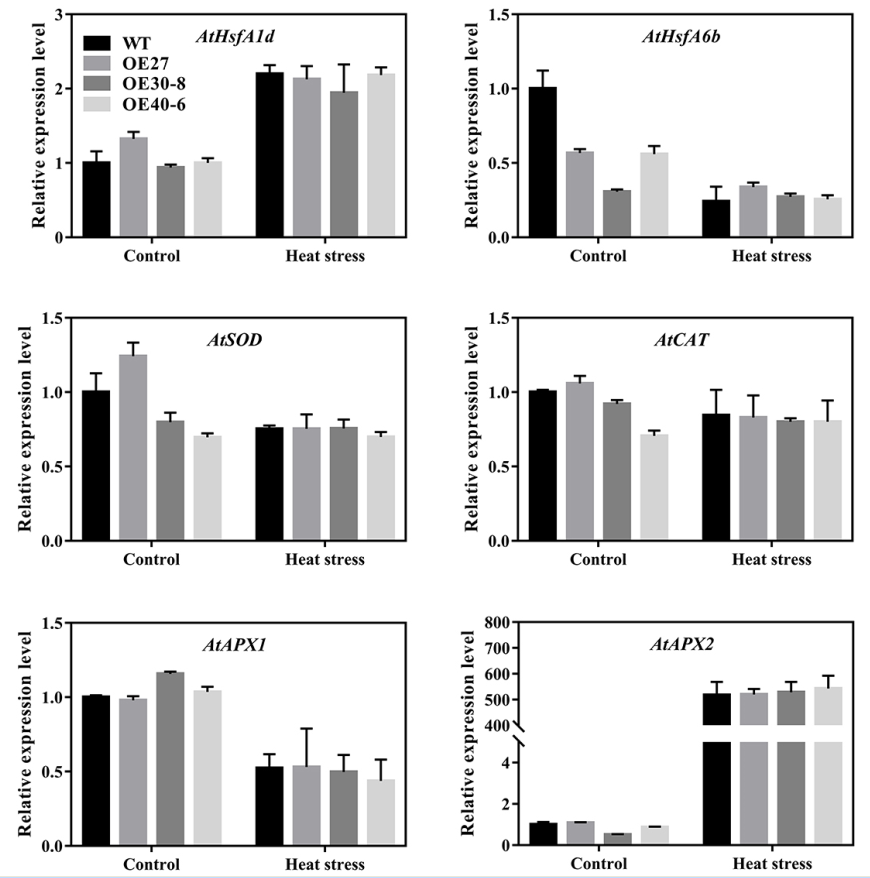


**Figure S3.** Expression of stress-related genes in *Arabidopsis*. The gene expression levels of 12-day-old *Arabidopsis* seedlings treated under 37°C for 0 or 2h were detected by qRT-PCR. All primers are shown in Table S1. Data are means ± SD of three biological replicates. The asterisks on the top of bars indicate significant differences between *CaHsfA1d* transgenic and WT plants. **P* < 0.05, ***P* < 0.01 by *t*-test. *AtHsfA1d* (At1G32330), *AtHsfA6b* (At3G22830), *AtSOD* (At1G08830), *AtCAT* (At1G20630), *AtAPX1* (At1G07890), *AtAPX2* (At3G09640).


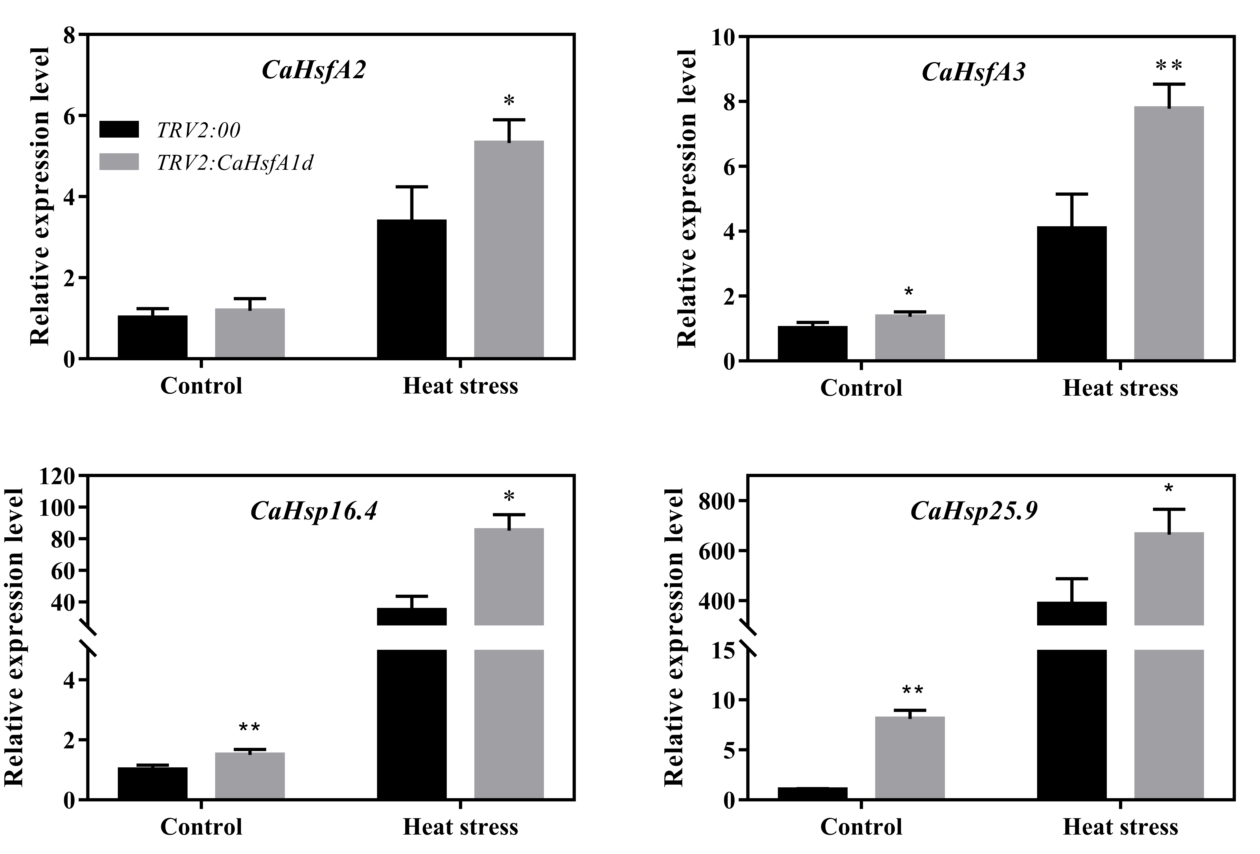


**Figure S4.** Expression of stress-related genes in pepper. The gene expression levels of *TRV2:00* and *TRV2:CaHsfA1d* seedlings treated under 42°C for 0 or 2h were detected by qRT-PCR. All primers are shown in Table S1. Pepper *CaUBI3* is used as a reference gene to normalize the transcript levels of *CaHsfA1d* upon different samples. Data are means ± SD of three biological replicates. The asterisks on the top of bars indicate significant differences between the *TRV2:00* and *TRV2:CaHsfA1d* plants. **P* < 0.05, ***P* < 0.01 by *t*-test.


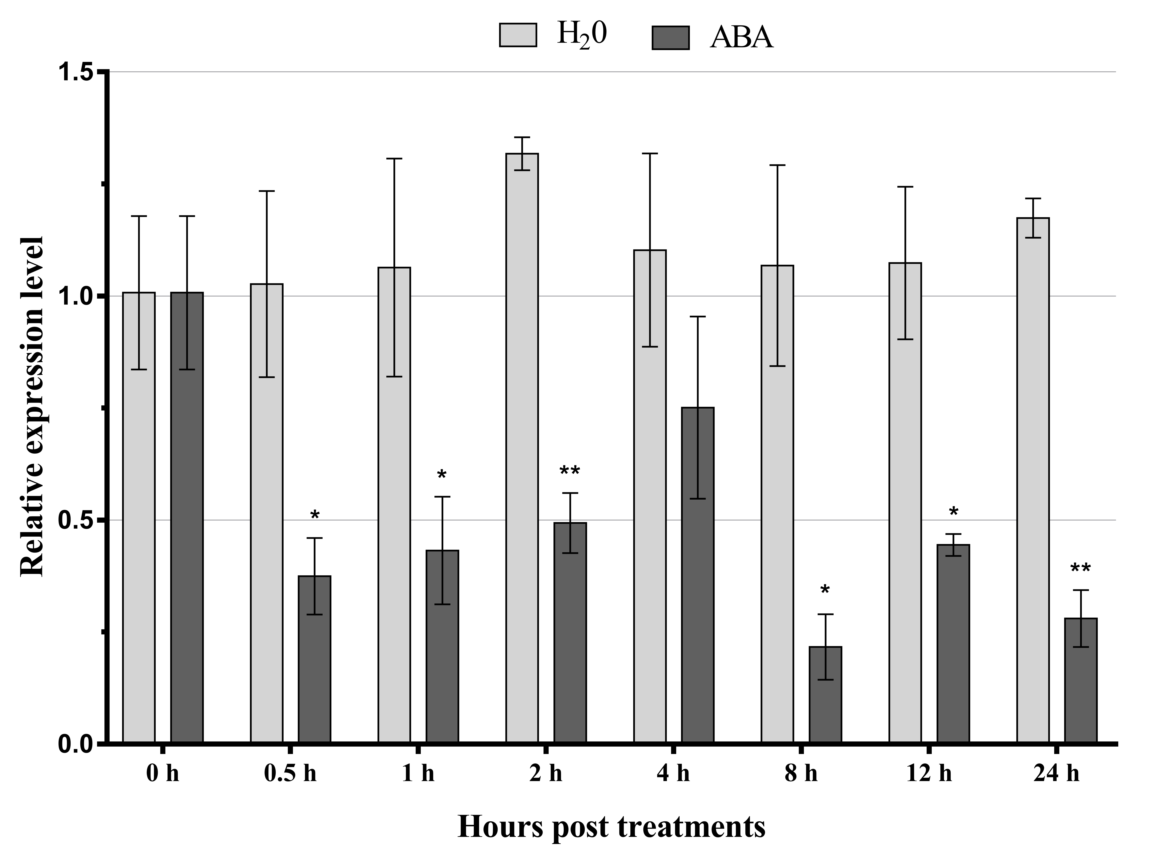


**Figure S5.** The *CaHsfA1d* expression in response to ABA treatment. 100 μM abscisic acid (ABA) solution was sprayed evenly onto the 6-8 true-leaf peppers. Control plants were mock-treated with H_2_O only. Pepper *CaUBI3* (ubiquitin-conjugating protein-coding gene) is used as a reference gene to normalize the transcript levels of *CaHsfA1d* upon different samples. The expression at 0 h is set as 1. Data are means ± SD of three biological replicates, each containing six seedlings. Different lowercases letters denote statistically significant differences from the control treatment at *p* ≤ 0.05 by *t*-test.

**Table S1** Primers used in this study

| Primer name | **Primer Sequence (5'→3')** |
| --- | --- |
| **For cloning** |  |
| CaHsfA1d-clone | F: ATGGGTTCTGCTTCAATGG  R: TACTTTTTTACTGTTTGATGTTAGTTG |
| **For vector construction** |  |
| BD-CaHsfA1d | F: ATGGCCATGGAGGCCGAATTCATGGGTTCTGCTTCAATGG  R: CTAGTTATGCGGCCGCTGCAGTACTTTTTTACTGTTTGATGTTAGTTG |
| BD-ΔNES | F: ATGGCCATGGAGGCCGAATTCATGGGTTCTGCTTCAATGG  R: CTAGTTATGCGGCCGCTGCAGGTTTTGAAGAAACTTTTCCCAG |
| BD-ΔAHA+ΔNES | F: ATGGCCATGGAGGCCGAATTCATGGGTTCTGCTTCAATGG  R: CTAGTTATGCGGCCGCTGCAGACCCACACTAGGAAGTTCTCC |
| BD-AHA+NES | F: ATGGCCATGGAGGCCGAATTCGATCCCTTCTGGGAAAAGTT  R: CTAGTTATGCGGCCGCTGCAGTACTTTTTTACTGTTTGATGTTAGTTG |
| BD-AHA | F: ATGGCCATGGAGGCCGAATTCGATCCCTTCTGGGAAAAGTT  R: CTAGTTATGCGGCCGCTGCAGGTTTTGAAGAAACTTTTCCCAG |
| pVBG2307-CaHsfA1d-GFP | F: GCTCTAGAATGGGTTCTGCTTCAATGG  R: GGGGTACCTACTTTTTTACTGTTTGATGTTAGTTG |
| TRV2-CaHsfA1d | F: GCTCTAGAGACGGTGGAAGTAACAATCA  R: GGGGTACCGTACTCTGCCACTCGGTTT |
| qRT-PCR-CaHsfA1d | F: GTGGGTGATCCCTTCTG  R: TTACTGTTTGATGTTAGTTGCT |
| sqRT-PCR-CaHsfA1d | F: GACGGTGGAAGTAACAATCA  R: GTACTCTGCCACTCGGTTT |
| **For RT-PCR** |  |
| AtHsfA1d | F: GGATTCAACACCAGTGGACAATG  R: AGGAGACCCATCTGTTGAGTCAG |
| AtHsfA2 | F: GTGTTGAGGTTGGGCAATACG  R: TTGCTGTTGCCTCAACCTAACTAC |
| AtHsfA3 | F: CCACCAGCAGCCTCAA  R: GTCATCCCTCCTAAACCCT |
| AtHSFA6b | F: GTGATGAAAGTGGTTATGGGAATG  R: TCCGACATCTCGAATTCAGACAT |
| AtDREB2A | F: GACCTAAATGGCGACGATGT  R: TCGAGCTGAAACGGAGGTAT |
| AtHSP15.7 | F: TGCCGGAGAATGTGAAAGTTG  R: CGATTTCGATGAAGTGTCCTTAGG |
| AtHsp26.5 | F: CAAAGAGTTATGGTTACTACAACACGA  R: ACGACACCGTATCTCTTCTACTCAA |
| AtHsp70b | F: TCCGCTTAGCCTTGGACTT  R: ACGCCTGGTTGATTGTCTG |
| AtHsp90.1 | F: GTGTTGGTTTCTACTCTGCTTAT  R: CCCCATCCACATCCCT |
| AtHsp101 | F: TGTCTTCAACACTCTGCTCCA  R: CACTTCCATTGTTACTTTCCCAG |
| AtGSTU5 | F: ATGGCTGAGAAAGAAGAAGTGAAGC  R: TTAAGAAGATCTCACTCTCTCTGCC |
| AtAPX1 | F: GTCCGACTCGCATGGCACTC  R: ACAACACCAGCAAGCTGATGGA |
| AtAPX2 | F: AATATGCTGCAGATGAGGATGC  R: CAAGAATCAAGGAGGTAGGAGATG |
| AtSOD1 | F: TCAACTGGAAATATGCAAGCGAGGT  R: ACCACACAGCTGAGTTGAGCAAA |
| AtCAT1 | F: AGCGCTTTCGGAGCCTCGTG  R: GGCCTCACGTTAAGACGAGTTGC |
| AtGPX3 | F: GGGTCAATCAGCGAGCTAC  R: CGATGGCGAAGAAGGGTATC |
| CaHsfA2 | F: AGCATCAGTAGCCACAGC  R: CTGCTTGATTCATTTTCCA |
| CaHsfA3 | F: TGGAAGGGCAGATAGTCA  R: AGAGCCATCGGGGTTA |
| CaHsp16.4 | F: ATGTCAAAGATGATCAGCTTATTG  R: GGGTACTCAACACGGGACAC |
| CaHsp25.9 | F: AGGAGACGACGCCTTAGTAG  R: TCTGACCCTTCTTTCTCTTCC |
