# Supplementary figures and images for: CaHsfA1d Improves Plant Thermotolerance via Regulating the Expression of Stress- and Antioxidant-Related Genes
Source: Int J Mol Sci. 2020 Nov 8;21(21):8374. doi: 10.3390/ijms21218374 (PMC7672572; doi:10.3390/ijms21218374)

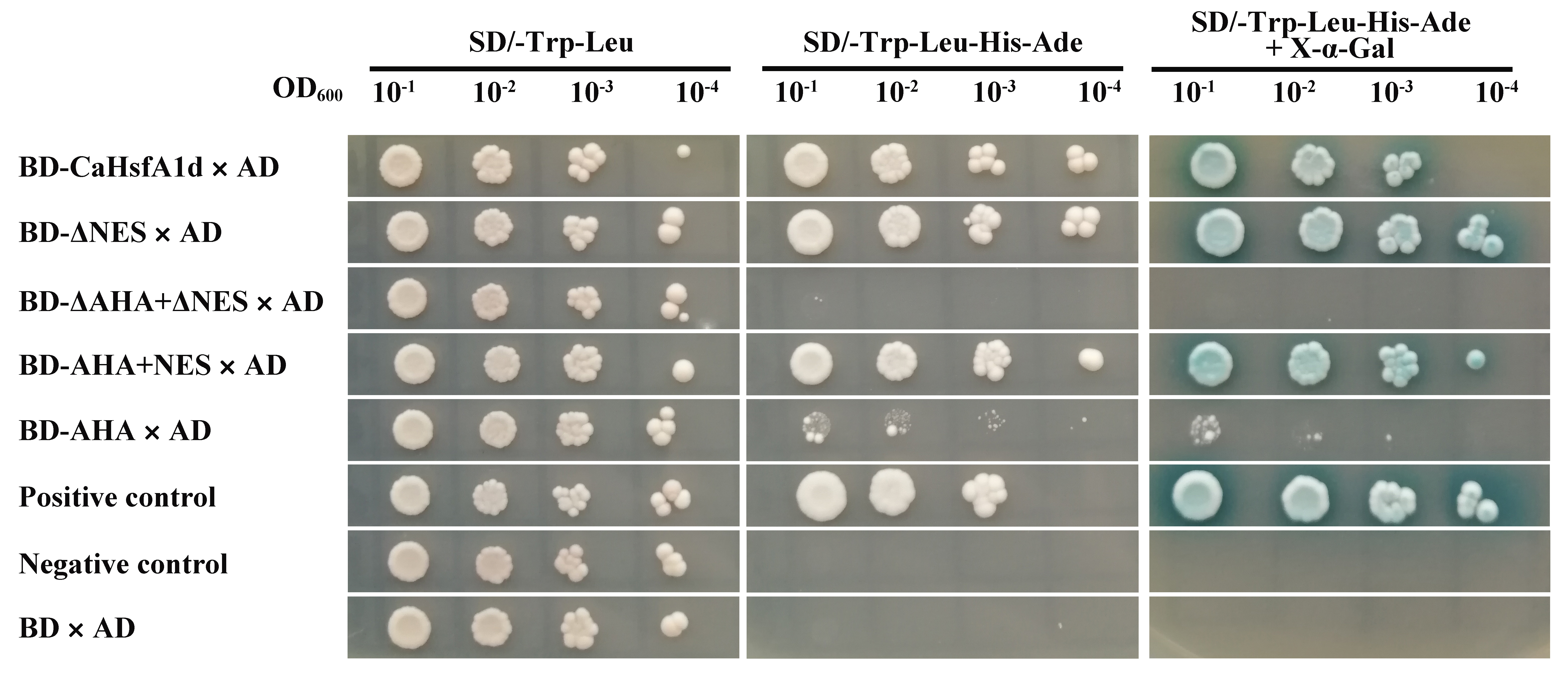

Supplement: Supplementary file 1 [file ijms-21-08374-s001.zip › sup/Figure S1.jpg]

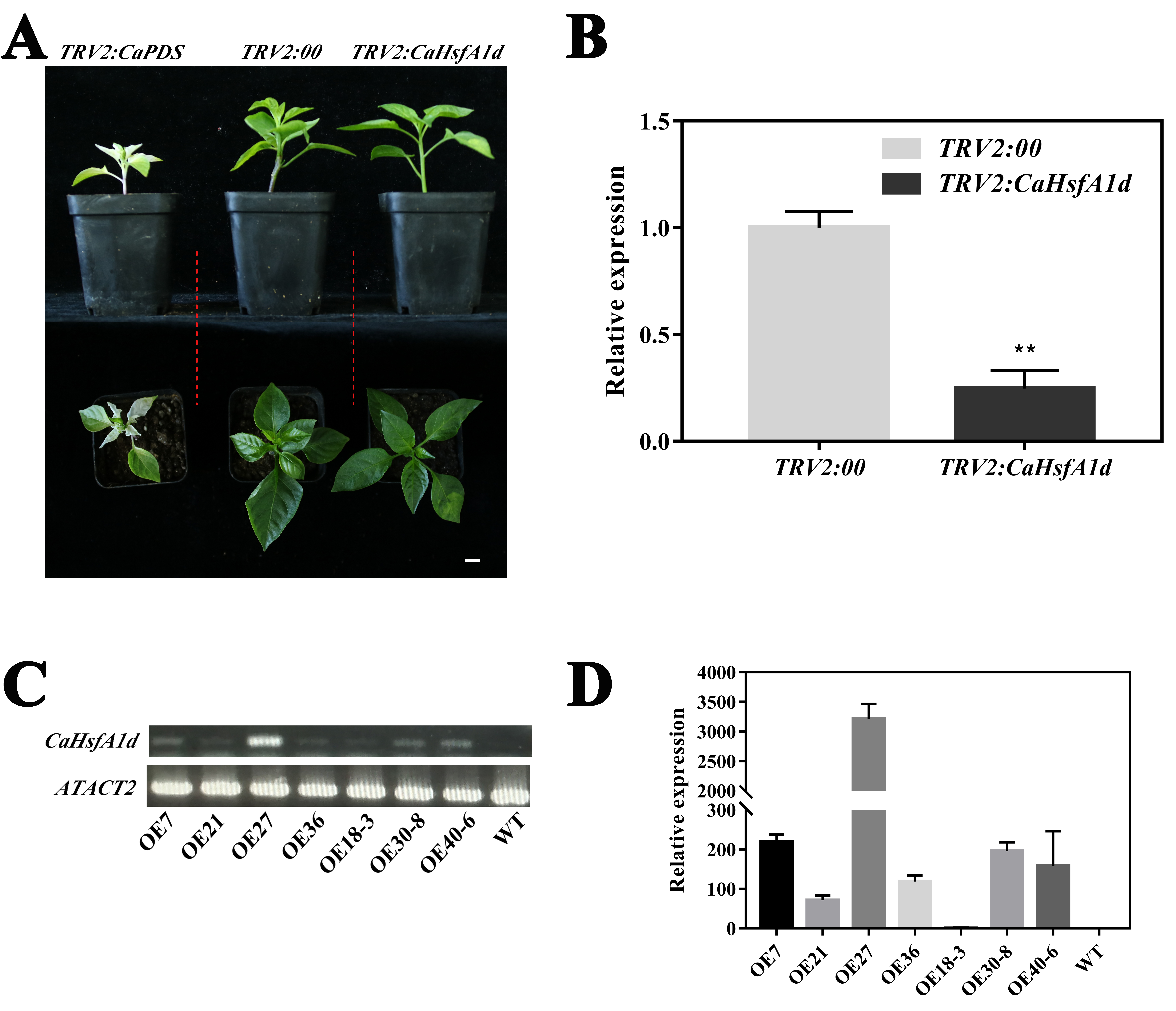

Supplement: Supplementary file 1 [file ijms-21-08374-s001.zip › sup/Figure S2.jpg]

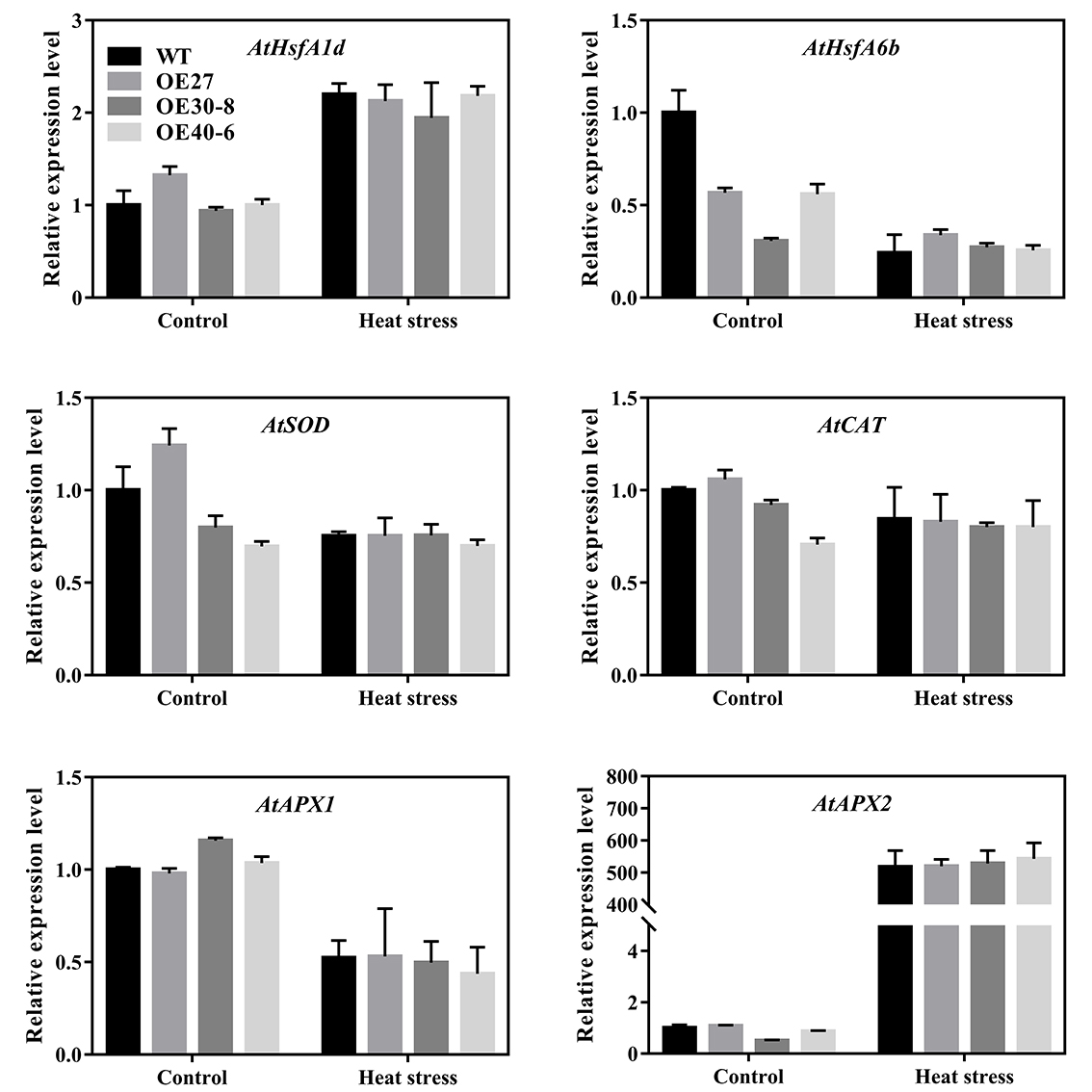

Supplement: Supplementary file 1 [file ijms-21-08374-s001.zip › sup/Figure S3.jpg]

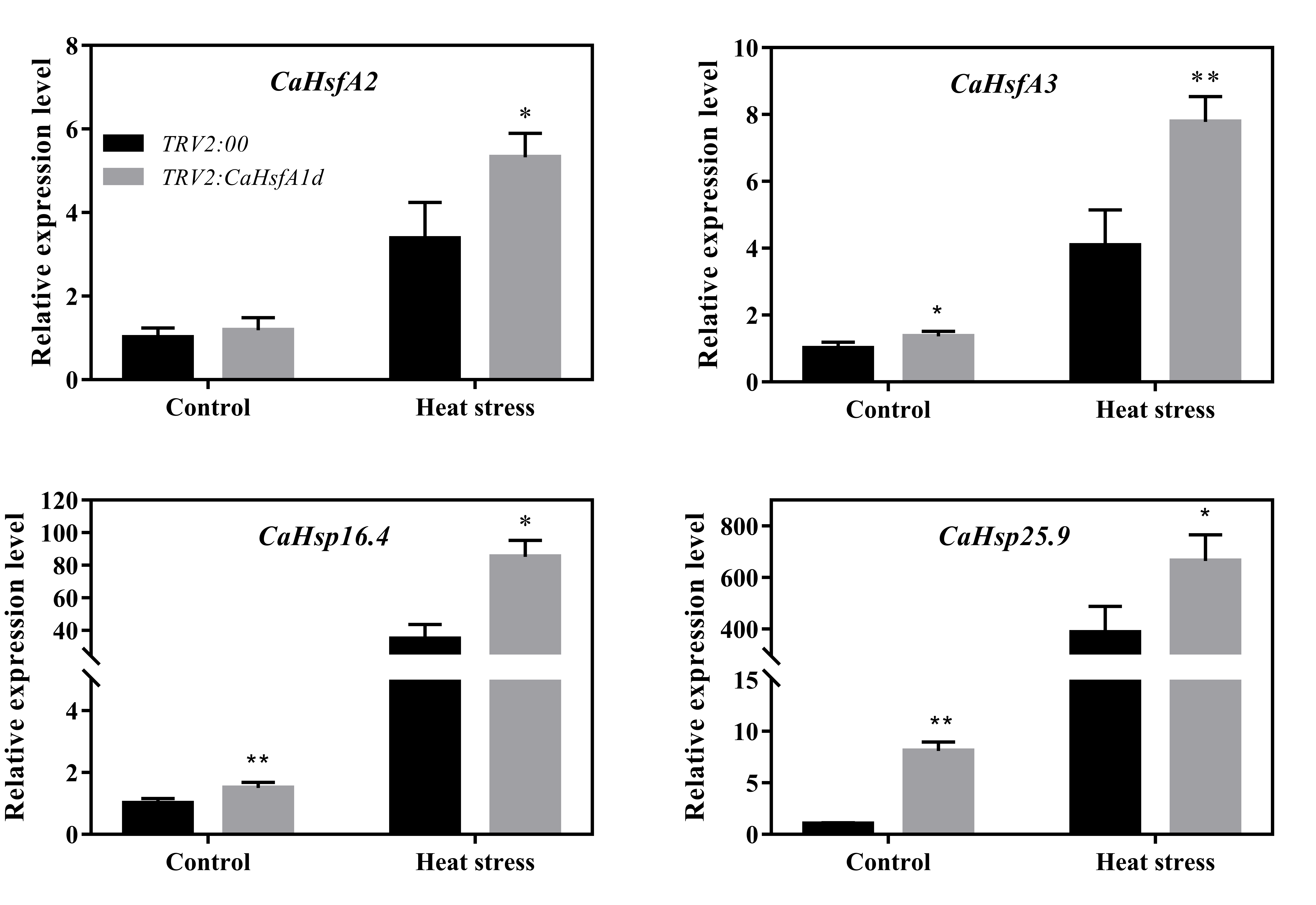

Supplement: Supplementary file 1 [file ijms-21-08374-s001.zip › sup/Figure S4.jpg]

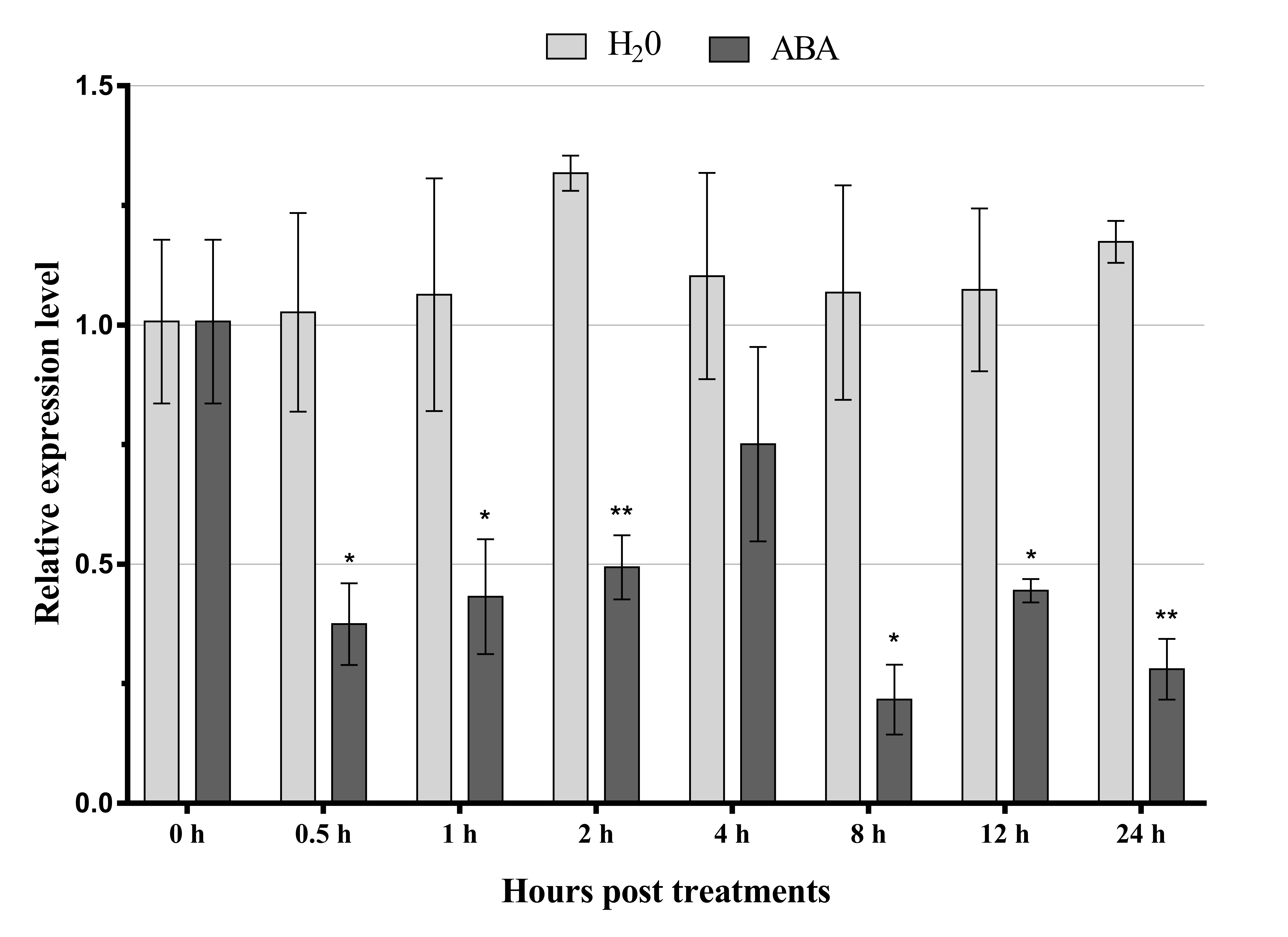

Supplement: Supplementary file 1 [file ijms-21-08374-s001.zip › sup/Figure S5.tif]
